# Attitudes toward milk alternatives and motives for (non-)consumption: an interview study with adolescents from Germany

**DOI:** 10.1186/s40795-025-01072-8

**Published:** 2025-05-26

**Authors:** Lena Szczepanski, Laura-Marie Bahlmann, Corinna Rötker, Annike Eylering, Florian Fiebelkorn

**Affiliations:** Biology Didactics, Department of Biology/Chemistry, Osnabrück University, Osnabrück, Germany

**Keywords:** Consumer research, Qualitative research, Sustainable nutrition, Dairy alternatives, Animal-free dairy, Young people, Germany

## Abstract

**Background:**

Consuming alternatives to cow’s milk (CM) could help counteract current environmental problems. Plant-based milk alternatives (PBMA) and precision fermentation-based milk alternatives–also called “animal-free milk” (AFM)–are potentially more sustainable alternatives to CM. Therefore, our study aimed to analyze young peoples’ attitudes toward PBMA and AFM and their motives for/against consuming these milk alternatives.

**Methods:**

For this purpose, semi-structured interviews were conducted with adolescents from Germany (*N* = 25; *M*_*Age*_ = 17.6). The data were analyzed using qualitative content analysis.

**Results:**

The results show that the young people in our study have positive attitudes toward PBMA, as they perceive PBMA as healthy and environmentally friendly. In addition, they have predominantly positive attitudes toward AFM, as they perceive AFM as an environmentally friendly and suitable alternative to CM. Animal welfare and environmental protection motivate the young people in our study to consume these milk alternatives. In contrast, sensory properties (PBMA) and food safety concerns (AFM) were identified as barriers to the consumption of milk alternatives.

**Conclusions:**

Adolescents generally have positive attitudes toward milk alternatives, motivated by perceived health, environmental, and animal welfare benefits. However, sensory properties and production-related uncertainties are barriers to consumption. These findings highlight the importance of addressing these concerns to foster the acceptance of sustainable milk alternatives among young people.

**Supplementary Information:**

The online version contains supplementary material available at 10.1186/s40795-025-01072-8.

## Background

Food production and livestock farming contribute significantly to current environmental problems, such as climate change and biodiversity loss [1–5]. Livestock farming accounts for approximately 60% of the anthropogenic greenhouse gas emissions of food production. It also contributes about 9% of total global CO₂ emissions and 40% of global anthropogenic methane emissions [5–7]. Furthermore, livestock farming requires 61–83% of the world’s farmland [4, 8, 9].

The consumption of cow’s milk (CM) remains consistently high worldwide and in Europe [10–12]. However, the recommended daily consumption amounts of milk and dairy products outlined in the national dietary guidelines of European countries vary greatly between 200 and 600 g [13]. In Germany, for example, milk is a staple food and an established element of people’s diets with a recommended consumption of two portions per day, which can be one slice of cheese (30 g) and one portion of milk (250 g) [14, 15]. Germany is also the largest milk producer in the European Union [16]. In 2022, about 32.4 million tons of CM were produced in Germany, and the average per-person consumption of CM in Germany has been around 50 kg per year over the last twenty years [16–18].

There are two potentially more sustainable alternatives to CM: Plant-based milk alternatives (PBMA) and “animal-free milk” (AFM) [1, 4, 19, 20]. PBMA are defined as suspensions of water and crushed or dissolved plant materials [21–23]. In general, PBMA are categorized according to whether they are made from cereals, legumes, nuts, oilseeds, or pseudo-cereals [21, 23–26]. PBMA, such as oat, soy, or almond milk, are intended to be similar to CM in appearance, sensory properties, and possible uses [22, 23]. The production of oat, soy, or almond milk causes three times less greenhouse gas emissions than the production of conventional CM, for example [1, 4]. However, the macro- and micronutrients in oat, soy, or almond milk do not correspond to those in CM, nor does the energy, fat, vitamin (e.g., B12), and other mineral (e.g., calcium) content [26–28]. Some producers thus enrich PBMA with calcium and/or vitamins [23].

In addition to continuously optimizing their PBMA portfolios, various companies are working on producing another milk alternative termed “animal-free milk” (AFM), which is equivalent to CM in appearance, sensory properties, and nutrients [20, 29–31]. Like CM, AFM mainly consists of water and the CM proteins casein and whey protein, which are produced via precision fermentation. During precision fermentation, microorganisms (e.g., fungi or yeasts) are genetically modified to express casein and whey protein, which are then mixed with minerals, sugar, water, and vegetable fats to produce the AFM [20, 29, 30, 32–34]. While the first alternative dairy products made with precision fermentation technology have been available in the United States, Singapore, and Hong Kong since 2021, these products are not yet available on the European market [34–36]. In Germany, only the food technology company Formo is working on the production of cheese alternatives (i.e., “animal-free cheese”) using precision fermentation technology [34, 37]. In general, information about the potential sustainability of dairy products made with precision fermentation technology should be considered carefully because companies are currently researching and developing animal-free dairy products and are thus unwilling to publish the details of their production processes [38]. Furthermore, compared to the production of conventional CM, the production of AFM is estimated to emit 35–97% less greenhouse gases and requires 77–97% less land [20, 35, 39, 40]. In addition to the lower environmental impact of AFM compared to conventional CM, its taste, appearance, and uses are equivalent to those of conventional CM, which could encourage people to make changes to their dietary habits by reducing their consumption of animal-based foods [31, 41].

In the European Union, 28% of all adults already consume PBMA [42, 43], while in Germany, 33–38% of people do [43, 44]. The most consumed PBMA in Germany are oat, soy, and almond milk [11, 45]. Whether people would be willing to consume AFM has not yet been investigated, though. To date, only the willingness to consume other animal-free dairy products, such as cheese, has been investigated [31, 32, 46–49]. For instance, a study with young adults from the United States showed that 64% of participants would be willing to buy products created with precision fermentation technology [46]. In Germany, Zollman Thomas and Bryant [31] showed that 75.9% of adult consumers would try animal-free cheese, 62.7% would buy it, and 36.1% would buy it regularly. To analyze consumer intentions and behaviors in relation to the consumption of milk alternatives, consumers’ attitudes and motives have often been examined in existing research [50–52].

### Attitudes toward milk alternatives

Attitudes are defined as an overall evaluation of an attitude object [53–55]. According to the ABC model of attitudes, they consist of three components: affective (A), behavioral (B), and cognitive (C) components. The affective component comprises a consumer’s feelings or emotional reactions to an attitude object (e.g., “Oat milk is delicious”). The behavioral component comprises a consumer’s behavior or behavioral intentions toward an attitude object (e.g., “I would never drink oat milk”). The cognitive component comprises a consumer’s beliefs about an attitude object, i.e., what they think about the attitude object in terms of fact (e.g., “Oat milk is healthier than CM”) [54, 55]. Consumers’ attitudes toward a product cannot be determined simply by identifying their beliefs [53, 54]. Understanding consumers’ attitudes toward milk alternatives could provide valuable insights into their decision-making processes, which can then be used to develop education and marketing campaigns [56].

The current research focuses on adults’ attitudes toward PBMA, mostly in comparison to CM [50–52, 56–60]. Overall, most studies have demonstrated that PBMA are perceived less positively than CM [50, 51, 57, 59]. Specifically, PBMA—in comparison to CM—are consistently evaluated as unhealthier, less nutritious, worse for the bones [50], and more expensive [51, 56]. To our knowledge, only two studies have investigated adolescents’ attitudes toward PBMA. Palacios et al. [61] showed that adolescents from the United States rated soy milk slightly more negatively than CM, whereby unflavored CM was rated less favorably than chocolate-flavored soy milk [61]. In Germany, a third of adolescents have exhibited a positive attitude toward PBMA [62].

Attitudes toward AFM have not yet been investigated. Broad et al. [32], Zollman Thomas and Bryant [31], and Zollman Thomas and Dillard [63] were the only studies that examined adults’ perceptions of animal-free dairy products. In the study by Zollman Thomas and Bryant [31], animal-free cheese was evaluated as less tasty and natural than conventional cheese. Zollman Thomas and Dillard [63] showed that potential consumers from the United States of America and Germany, for example, perceived the production of animal-free dairy products as technical and scary. However, animal-free cheese was evaluated as the most ethical and environmentally friendly product compared to conventional and vegan cheese. Among German adults, the perception of animal-free cheese as ethical, tasty, and healthy positively affected their willingness to consume animal-free cheese [31]. To our knowledge, young people’s attitudes toward AFM or dairy products have not yet been investigated.

### Motives for/against the consumption of (or willingness to consume) milk alternatives

In addition to attitudes toward a product, motives play a decisive role in consumers’ decision-making as they affect attitudes [64, 65]. In general, motives are defined as a person’s reasons for a specific behavior [66]. Therefore, consumers’ motives in food selection correspond to their goals: Motives describe what the consumer wants to achieve in or with their food consumption [54]. Food choice motives influence consumers’ attitudes toward a product [50, 64]. Consumers are often unaware of their food choice motives. However, they can be made aware of their motives through reflection or questioning [67]. Insights into consumers’ food choice motives can be used to develop and optimize marketing strategies by addressing (potential) consumers’ motives in marketing communication to increase their consumption of (or willingness to consume) certain products [54]. In addition to physiological factors, health, convenience, natural content, price, weight control, familiarity, and ethical concerns are motives that affect food choices. Furthermore, consumer behavior can be determined by social motives [65]. For European consumers, price, sensory appeal, and natural content are the most important food choice motives [68].

Regarding the consumption of milk alternatives, current research focuses on adults’ motives for and against the consumption of PBMA [44, 51, 52, 57–59, 69–73]. Adults from the United States of America, Austria, and Germany, for example, often cite health, environmental, and ethical aspects as motives for consuming PBMA [50–52, 57–59, 69, 73]. Adults in Germany indicate specific diets and health reasons, as well as environmental and animal protection as motives for not consuming milk and dairy products [44].

Motives for and against the consumption of AFM have not yet been investigated. However, a few studies exist that examine adults’ motives for their willingness to consume animal-free dairy products. Animal welfare and environmental protection have been identified as motives for the willingness to consume animal-free dairy products among adults. Uncertainties regarding food safety were identified as motives against the willingness to consume animal-free dairy products [32, 46, 63].

### Study aims and research questions

To foster a change in dietary habits, we must analyze the consumption of or willingness to consume more sustainable milk alternatives. Attitudes and motives offer a route to analyze this consumption or willingness to consume. The existing research has focused on adults’ attitudes toward PBMA and their consumption motives. Attitudes toward PBMA have often been investigated quantitatively via attitude statements or semantic differentials to determine consumers’ perceptions. To date, limited research has been conducted on the specific beliefs and feelings about the consumption of milk alternatives that underlie positive or negative attitudes toward them. In addition, motives for or against the consumption of PBMA have rarely been surveyed compared to the current consumption of PBMA.

Therefore, the first aim of our study is to identify how PBMA and AFM are evaluated, and which beliefs and feelings determine a positive or negative evaluation. The second aim of our study is to determine the motives for and against the consumption of (or willingness to consume) PBMA and AFM based on the current consumption (or willingness). A qualitative research approach was chosen to allow participants to express and explain their beliefs, feelings, and motives for consuming or being willing to consume milk alternatives [67, 74].

In this study, we focus on adolescents in Germany, as they have not been investigated and represent a crucial group for shaping future dietary trends as their dietary habits are less entrenched and easier to influence than those of adults [75, 76].

This results in the following research questions:

Research Question 1: What are adolescents’ attitudes toward milk alternatives?

 Research Question 1.1: How do adolescents evaluate PBMA?

 Research Question 1.2: How do adolescents evaluate AFM?

Research Question 2: What are adolescents’ motives for/against the consumption of (or willingness to consume) milk alternatives?

 Research Question 2.1: What are adolescents’ motives for/against the consumption of PBMA?

 Research Question 2.2: What are adolescents’ motives for/against their willingness to consume AFM?

## Methods

The methods are described following the “Qualitative Design Reporting Standards (JARS-Qual)” [77] and the criteria for reporting qualitative research (COREQ) [78].

### Selection of participants, characteristics of the participants, and recruitment

For sample construction, the inductive sampling method for exploratory studies according to Reinders [79] was chosen. The sample was selected based on three criteria. Firstly, adolescents needed to attend upper secondary school because they need biological knowledge about genetics to understand the production of AFM, which is covered in the upper secondary school biology curriculum in Germany [80]. Secondly, the adolescents needed to identify as different genders, and, thirdly, consume CM or PBMA to allow us to identify their attitudes toward milk alternatives and their motives for and against consumption across genders (female, male, and other) and consumer groups (CM consumers and PBMA consumers).

The final sample consisted of 25 adolescents 16 to 19 years old (*N* = 25; *M*_*Age*_ = 17.60 years; *SD*_*Age*_ = 0.63 years; 60% female). The adolescents came from five secondary schools in the city and district of Osnabrück (Lower Saxony, Germany) and were in the eleventh to thirteenth grades (upper secondary school) at the time of data collection. Descriptive statistics of the sample and their self-reported consumption of CM, PBMA, and their willingness to consume AFM are presented in Table 1. All names have been replaced with pseudonyms to ensure anonymity.

**Table 1:** Overview of the sample of adolescents (*N* = 25)

Name ^a^	Gender	Age	Residence ^b^	Interview duration	Diet ^c^	Consumption CM ^d^	Consumption PBMA ^e^	Willingness to consume AFM ^f^
Liam	male	17	urban area	50.30 min	omnivorous	daily	never	willing
Sophia	female	17	rural area	43.11 min	omnivorous	weekly	casual (monthly)	willing
Alexander	male	17	urban area	47.63 min	omnivorous	daily	never	not willing
Emma	female	17	rural area	45.11 min	vegetarian	monthly	regular (daily)	willing
Elias	male	18	rural area	39.01 min	vegetarian	monthly	casual (monthly)	not willing
Mathys	male	17	rural area	29.33 min	omnivorous	daily	never	willing
Mason	male	17	urban area	46.80 min	omnivorous	weekly	regular (daily)	willing
Benjamin	male	16	urban area	41.13 min	other	weekly	casual (weekly)	willing
Oliver	male	17	rural area	41.40 min	other	never	regular (weekly)	willing
Emilia	female	17	rural area	32.32 min	vegetarian	monthly	regular (weekly)	willing
Mila	female	18	rural area	38.01 min	omnivor	daily	never	willing
Lily	female	18	rural area	35.07 min	vegetarian	weekly	regular (daily)	willing
Sophie	female	18	urban area	40.47 min	omnivorous	never	casual (weekly)	willing
Mia	female	19	urban area	49.21 min	vegetarian	monthly	regular (daily)	willing
Isabella	female	18	rural area	50.67 min	omnivorous	daily	never	willing
Maëlys	female	18	urban area	42.56 min	vegetarian	weekly	never	willing
Jack	male	18	rural area	34.73 min	omnivorous	daily	regular (daily)	willing
Samuel	male	18	urban area	46.07 min	omnivorous	never	casual (weekly)	willing
Ella	female	18	urban area	51.63 min	omnivorous	weekly	casual (weekly)	willing
Olivia	female	18	rural area	47.87 min	vegetarian	weekly	casual (weekly)	willing
Emmy	female	17	urban area	46.07 min	vegan	never	regular (weekly)	willing
Amelia	female	18	rural area	39.88 min	omnivorous	daily	never	willing
Charlotte	female	18	urban area	35.73 min	omnivorous	daily	never	willing
Emily	female	18	rural area	35.78 min	omnivorous	weekly	regular (weekly)	willing
Lucas	male	18	urban area	37.63 min	omnivorous	daily	regular (daily)	willing
Mean value		17.60		41.86 min				
Standard deviation		00.63		06.07 min				
Minimum		16.00		29.33 min				
Maximum		19.00		51.63 min				

*Notes*. ^a^ Randomly selected pseudonym according to the most popular first names in 2022 in the United States of America. ^b^ The adolescents were asked about their area of residence after the interview and could choose either urban area or rural area. ^c^ The adolescents were asked about their diets after the interview and could choose omnivorous, vegetarian, vegan, or other. ^d^ The adolescents were asked about their consumption of CM after the interview and could choose daily, weekly, monthly, or never. ^e^ During the interview, the adolescents were asked to describe their consumption of PBMA and, on this basis, classify their consumption as regular, casual, or never. The frequency of consumption is given in parentheses. ^f^ During the interview, the adolescents received an informative text about AFM and were asked to decide accordingly whether they were willing or unwilling to consume AFM.

To recruit participants, the school principals of all secondary schools in the city and district of Osnabrück were contacted by email with an invitation to participate in this study. The school principals forwarded the email to the biology teachers at their schools and asked the biology teachers to request voluntary participants from their upper secondary school courses. The biology teachers were instructed to ensure an equal distribution of genders and consumption of PBMA and CM when selecting participants.

The interviews were always scheduled in blocks by the teachers so that the participants from the same school were interviewed on the same day and one after the other. After each interview block, the first and third authors (two coders) discussed content saturation. The coders used the deductive category system as a basis and the audio recordings to discuss whether new content and inductive codes had been added. Content saturation occurred after 21 interviews. At this point, four more interviews were planned, which were subsequently conducted and a total of 25 interviews were analyzed.

Before the data were collected, both the school principals and the students (and their parents, for students under the age of 18) signed a declaration of consent. The student’s parents also received an informative letter explaining the study’s aim, that participation was voluntary, and that the data would be anonymized. Before the interviews began, students’ (and parents’) signed declaration of consent was reviewed and confirmed verbally. Participation in the interviews was voluntary and anonymous. All students had the opportunity to decline participation at any time without consequences.

Data collection occurred in September and October 2022. The first author (characteristics: female; qualification: physics and biology teacher with two years of teaching experience; current occupation: PhD student and research assistant) conducted all interviews in a quiet room in the students’ schools. The interviews were recorded with a digital audio recorder (Olympus LS-P1). They ranged from 29.33 to 51.63 min (*M*_*Duration*_ = 41.83 min; *SD*_*Duration*_ = 6.07 min).

### Study design and interview procedure

We chose a qualitative approach with in-depth semi-structured interviews to better understand adolescents’ attitudes and motives in relation to the consumption of PBMA and AFM since qualitative methods allow for a detailed investigation of individual associations, beliefs, and feelings, which are difficult to assess through standardized questionnaires [64]. For our study, the first author, in cooperation with biology didactics experts, biology teachers, and psychologists, developed an interview guide based on (consumer) psychological theories [53, 54, 64]. We decided to use a semi-structured interview guide to allow for more flexibility and to enable participants to express their thoughts more freely [79]. To gain a deeper understanding of participants attitudes and consumption motives, we mainly asked “why” questions [54, 64]. To optimize the interview questions and process, the interview guide was tested in a pilot study with four students aged 17 to 18 before data collection [81, 82]. Minimal adjustments were made to the questions based on the pilot study results. Notably, the pilot interviews were not included in the final sample.

**Fig. 1:**
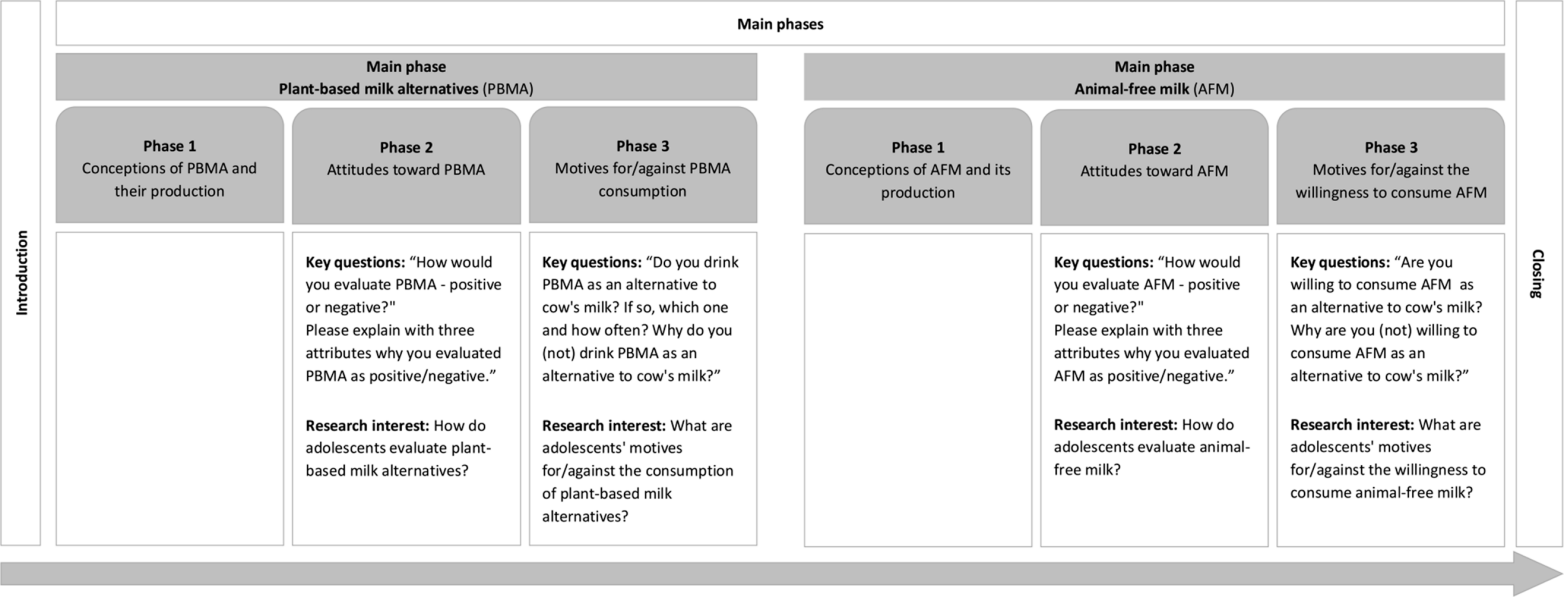
*Key questions and research interests in the main phases of the interviews about milk alternatives* *Notes*. This study did not analyze phase 1 of the main phases of the interview. In the first phase, the adolescents were asked about their conceptions of PBMA and AFM and their production

Interviews were divided into four broad phases: (1) the introduction, (2) the main phase on PBMA, (3) the main phase on AFM, and (4) the conclusion with a short questionnaire (Fig. 1). During data collection, the order of the second and third phases was switched after each participant to minimize any possible influence of the order of the phases on participants’ responses. The second and third phases were divided into three subphases: (1) conceptions of PBMA/AFM and their/its production, (2) attitudes toward PBMA/AFM (RQs 1.1 and 1.2), and (3) motives for and against the consumption of PBMA/AFM (RQs 2.1 and 2.2; Fig. 1). To answer the research questions, only data on attitudes and motives from the second and third phases were analyzed. The complete interview guide, including guiding questions and prompts, is provided in the supplementary material (Supplementary Material 1).

Before the first phase, the interviewer talked with the participant about how they were doing and briefly introduced herself (a PhD student at a university researching “sustainable nutrition”). The interviewer clarified that the interview topic was “milk alternatives” and pointed out that the interview was not about their knowledge but their thoughts on the topic. Participants were also informed that the collected data would be treated confidentially, meaning that their teachers would not receive any of the data, and they were encouraged to say anything that came to mind.

During the first phase, participants were asked about their conceptions of PBMAs and AFM, their ingredients, and their production; this phase is not included in this article. Additionally, participants were shown the list of ingredients for a carton of oat drink (water, oats [12%], sunflower oil, and sea salt) so that they could discuss their conceptions of the production of PBMAs. After this first phase, participants were given no further information about the production of PBMAs.

During the second phase, participants were asked to evaluate PBMAs or AFM overall as “(rather) positive” or “(rather) negative” to assess their attitudes toward milk alternatives [53]. Participants were then asked to explain their positive or negative evaluation using three product-related attributes to identify the beliefs and feelings about PBMAs or AFM that determined their positive or negative evaluation [55]. Since AFM is not yet available on the European market and the public is less familiar with dairy products produced with precision fermentation technology [31, 32], participants were given a brief informative text detailing what AFM is and how it is produced to help them evaluate it (Supplementary Material 2).

During the third phase, participants were first asked about their consumption of PBMA or their willingness to consume AFM. Their current consumption of PBMA was assessed by asking, “Do you drink PBMA as an alternative to CM?” If participants already consumed PBMA, they were asked to provide the frequency of consumption and types of PBMA consumed during a typical school week. In addition, participants were asked to classify their consumption of PBMA as “regular,” “casual,” or “never” based on their self-reported consumption frequency. Participants were also asked whether they would be willing to consume AFM as an alternative to CM if it became available in all supermarkets and restaurants. Based on their responses to this question, participants’ motives for and against the consumption of milk alternatives were assessed with the questions, “Why do you never/casually/regularly drink PBMA as an alternative to CM?” and “Why are you (not) willing to consume AFM as an alternative to CM?” [64]. During the interview, the interviewer asked individual, thought-provoking questions to identify participants’ feelings, beliefs, and thoughts about the topic (see Supplementary Material 1). At the end of the interview, participants were asked to complete a short questionnaire on their sociodemographic data and consumption of milk (alternatives; Supplementary Material 1).

### Data analysis

The audio recordings were transcribed according to Dresing and Pehl [83]. The transcripts were not returned to the participants for comments or corrections. In the next step, the transcripts were redacted according to Gropengießer’s [84] guidelines to improve readability and reduce the research question content in the transcripts. The transcription and redaction rules are provided in the supplementary material (Supplementary Material 3). The redacted data were analyzed in MAXQDA [85] with qualitative content analysis according to Kuckartz and Rädiker [86] which enables a systematic but flexible analysis of qualitative data through a combination of deductive and inductive coding.

As we followed a qualitative interview approach, we do not have a representative sample of adolescents in Germany. Therefore, we do not intend to make generalized statements for adolescents (in Germany) with our qualitative results. As part of the content analysis, we use frequencies to illustrate trends within our sample [77].

**Fig. 2:**
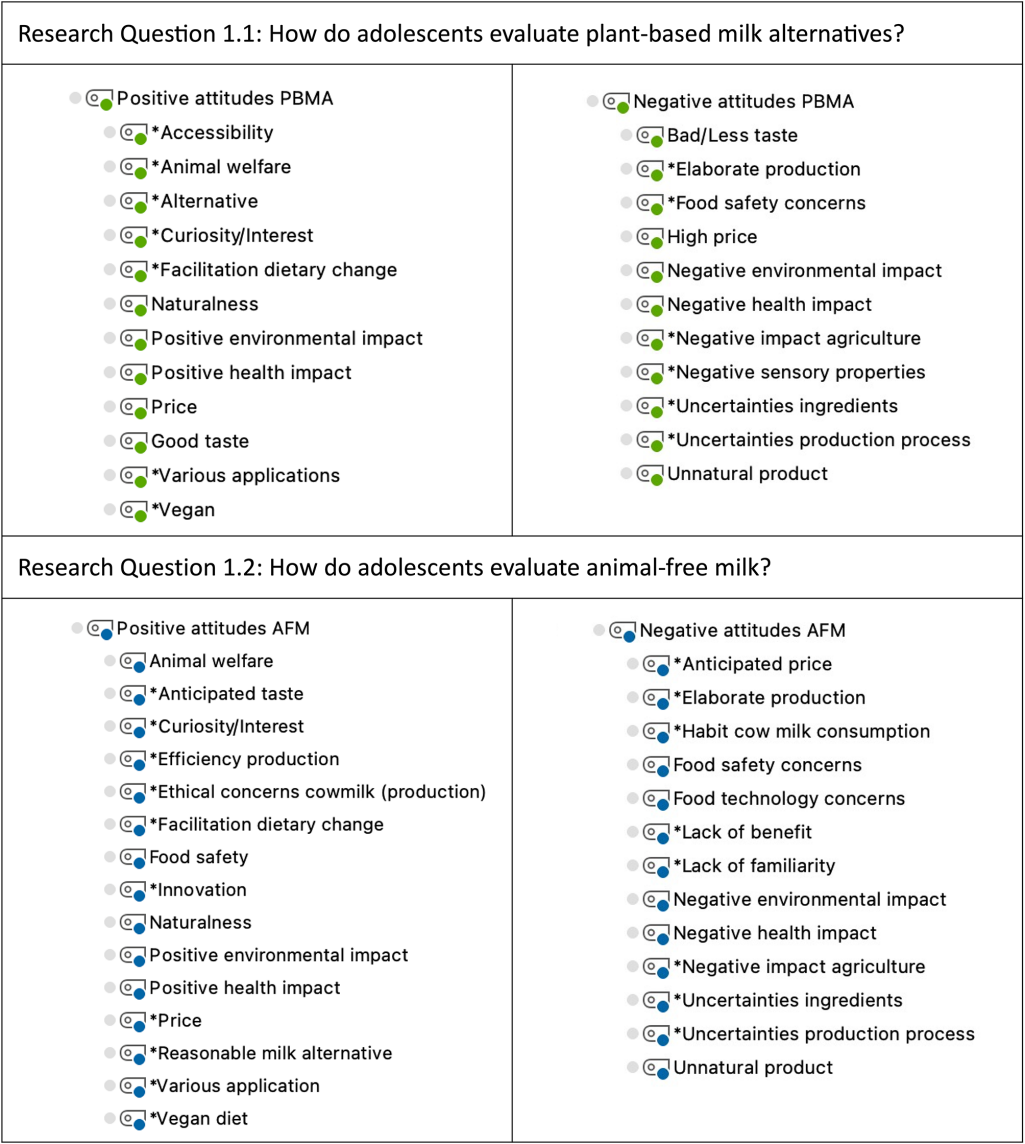
*Category systems for analyzing adolescents’ attitudes toward milk alternatives* *Notes*. * Inductively coded categories. PBMA = plant-based milk alternatives; AFM = animal-free milk

**Fig. 3:**
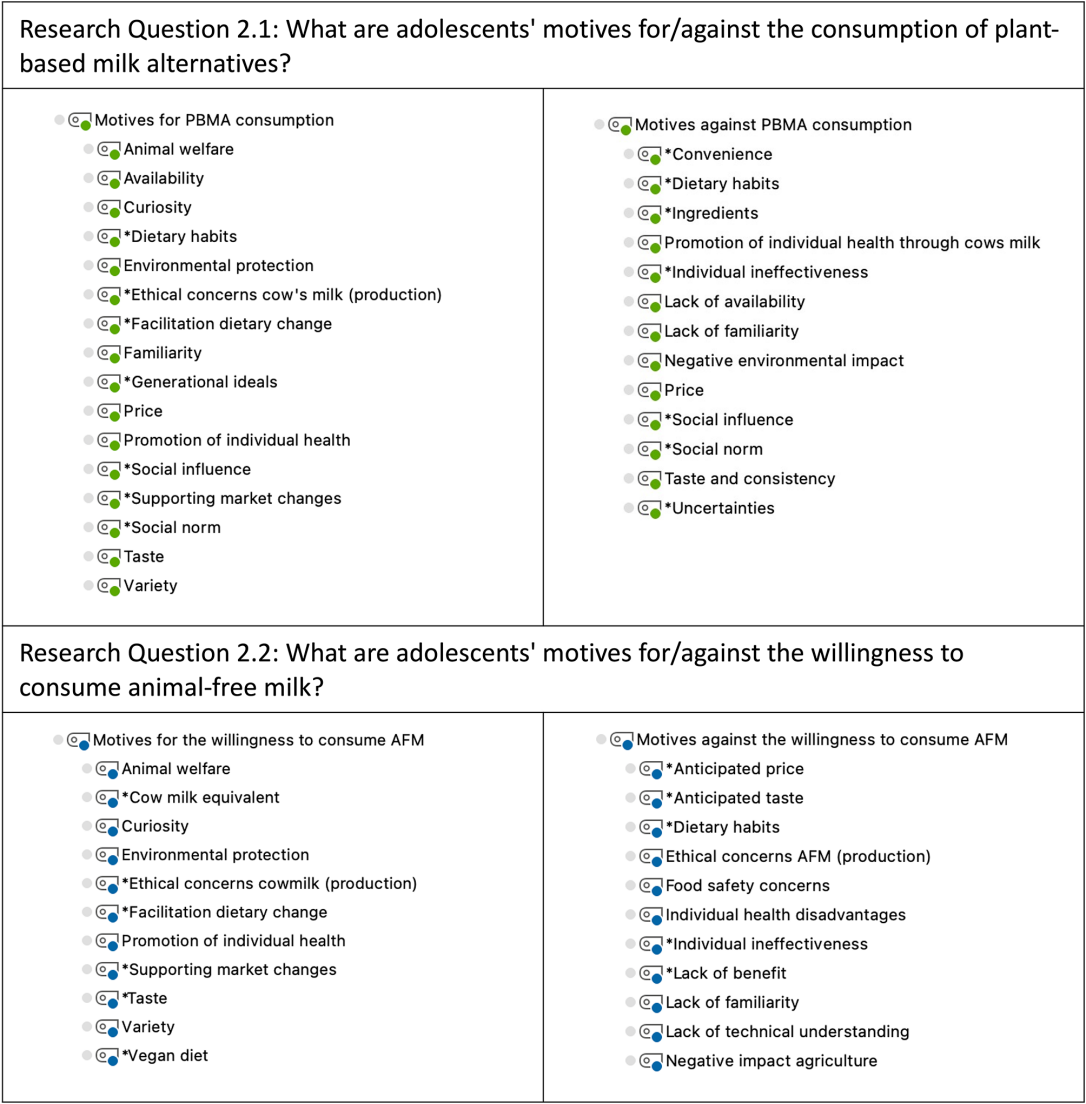
*Category systems for analyzing adolescents’ motives for and against the consumption of (or willingness to consume) milk alternatives* *Notes*. * Inductively coded categories. PBMA = plant-based milk alternatives; AFM = animal-free milk

According to the research questions and the interview phases, a deductive category system was created with the following superordinate categories: (1.1) “Attitudes toward PBMA,” (1.2) “Attitudes toward AFM,” (2.1) “Motives for/against PBMA consumption,” and (2.2) “Motives for/against the willingness to consume AFM” (Figs. 2 and 3). The superordinate categories “Attitudes toward PBMA” and “Attitudes toward AFM” were each deductively divided into two subcategories, “Positive attitudes toward PBMA/AFM” and “Negative attitudes toward PBMA/AFM”. Within each of these subcategories, deductive codes were derived from the current state of research, and inductive codes (*) were derived from participants’ statements during the data analysis (Fig. 2).

The superordinate categories “Motives for/against PBMA consumption” and “Motives for/against the willingness to consume AFM” were also each deductively divided into two subcategories (“Motives for PBMA consumption/the willingness to consume AFM” and “Motives against PBMA consumption/the willingness to consume AFM”). Within each of these subcategories, deductive codes were derived from the current state of research, and inductive codes (*) were derived from participants’ statements during the data analysis (Fig. 3).

The complete category system is provided in the supplementary material (Supplementary Material 4). The analysis of the entire qualitative data was carried out by the first author. To affirm the internal consistency of the coding, 20% of all statements that were relevant for answering the research questions were also coded with the complete category system by the second author [86]. To assess intercoder agreement, the kappa coefficient was calculated in MAXQDA according to Brennan and Prediger [87]. The kappa value is 0.79, which indicates substantial intercoder agreement [87].

## Results

In total, seventeen of the 25 adolescents stated they already consumed plant-based alternatives to CM. Of these, seven stated that they consumed PBMA casually, and ten regularly. The regular consumers in our study said that they used PBMA three to seven days a week, while the casual consumers used PBMA two to four days a week. The regular and casual consumers reported that they most frequently consumed oat milk (*n* = 15), followed by almond milk (*n* = 5). Eight of the adolescents stated that they did not consume any plant-based alternatives to CM. Of these, six said that they had not yet tried any PBMA (Table 1). Concerning AFM, 23 of the adolescents said that they would be willing to try, buy, or drink AFM as an alternative to CM. Two adolescents stated that they would not be willing to try AFM (Table 1).

### Attitudes toward PBMA

Overall, all adolescents in our study (*N* = 25) evaluated PBMA as positive. The attributes that the participants mentioned most frequently to justify their positive evaluation of PBMA were assigned to the categories “Positive health impact,” “Positive environmental impact,” and “Alternative” (Table 2). The adolescents in our study most frequently justified their positive evaluation with the belief that PBMA are as healthy or healthier than CM (*n* = 19) and more environmentally friendly than CM (*n* = 17). Specifically, they argued that PBMA are nutrient-rich, lactose-free, low in fat and sugar, and can only be healthy due to their plant-based ingredients. In terms of the anticipated positive environmental impact, they argued that greenhouse gas emissions from the production of PBMA are lower than those from the production of CM and that fewer resources, such as land and water, are required. As a further justification for their positive evaluation, the adolescents mentioned the availability and use of PBMA as alternatives to CM, especially for people with lactose intolerance (*n* = 13). The participants also mentioned attributes from the following categories to justify their positive evaluation of PBMA: “Good taste” (*n* = 12), “Animal welfare” (*n* = 10), “Various applications” (*n* = 7), “Curiosity/Interest” (*n* = 5), “Vegan” (*n* = 4), “Naturalness” (*n* = 2), “Accessibility” (*n* = 2), and “Facilitating dietary change” (*n* = 1; Supplementary Material 5).

**Table 2:** *Top three categories and attributes for the positive evaluation of PBMA*

Positive attitudes toward PBMA ^a^			
Category	Attributes	Example quote ^b^	Frequency, *n* (%) ^c^
Positive health impact	healthy, low-fat	*“I think the milk alternative is healthier because* *oats*,* for example*,* are very healthy. If milk is made from it*,* […] it’s very healthy.”* (Jack, RC) *“In terms of health*,* I simply believe that the fat content is lower than in CM.”* (Sophie, CC) *“PBMA are healthy because when you think of plants*,* it’s always healthier than […] animal products.”* (Mila, NC)	19 (20.7)
Positive environmental impact	environmentally friendly, climate neutral	*“Cows emit a lot of methane. That’s very bad for the environment and they need a lot of feed. That’s why PBMA are positive for me […] and the terms environmentally friendly and ecological came into my head.”* (Jack, RC) *“PBMA are more CO*_*2*_*-neutral*,* as they perform* *better than CM in terms of CO*_*2*_*emissions.”* (Benjamin, CC) *“PBMA are more environmentally friendly because keeping animals is very laborious and requires a lot of water.”* (Mathys, NC)	17 (18.5)
Alternative	alternative, substitutional	*“I would rate PBMA positively in principle because they really offer a different way […] to drink something dairy-like.”* (Mason, RC) *“For me*,* PBMAs are diverse and […] also an alternative*,* as you can use them for almost anything.”* (Ella, CC) *“PBMA are a good alternative […] that lactose intolerant people can fall back on.”* (Charlotte, NC)	13 (14.1)

*Notes.*^a^ Positive overall evaluation of PBMA (*n* = 25). ^b^ Statements from all adolescents who regularly (RC), casually (CC), or never (NC) consumed PBMA to justify their positive evaluations. ^c^ Percentage of all coded segments in the category “Positive attitudes” (*N* = 92)

In addition to their overall positive evaluation, the adolescents in our study evaluated PBMA negatively in some respects, such as environmental impact (*n* = 11), price (*n* = 6), and taste (*n* = 5). They argued that the production of some PBMA, such as soy milk, is not sustainable as the plant-based raw materials are not grown in Germany and their cultivation requires significant resources. As Isabella and Emily explained:

*“Another negative aspect is that rainforests have to be deforested to produce PBMA […].”* (Isabella, a non-consumer of PBMA)

*“If you look at […] soy*,* for example*,* I’ve often heard that soy is not grown in Germany*,* but in South America. If soy is flown over here for PBMA*,* I don’t think that’s very sustainable.”* (Emily, a regular consumer of PBMA)

In addition, the adolescents in our study evaluated the price and taste of PBMA negatively. They explained that they perceived PBMA as “too expensive” and “less tasty” than CM. Attributes of the following categories were also mentioned to justify their negative evaluation of PBMA: “Negative health impact” (*n* = 4), “Elaborate production” (*n* = 2), “Uncertainties regarding ingredients” (*n* = 2), “Food safety concerns” (*n* = 1), “Negative impact on agriculture” (*n* = 1), “Negative sensory properties” (*n* = 1), and “Uncertainties regarding the production process” (*n* = 1; Supplementary Material 5).

### Attitudes toward AFM

Overall, 22 of the 25 adolescents in our study evaluated AFM positively and three evaluated it negatively. To justify their positive evaluation of AFM, the adolescents most frequently mentioned attributes in the categories “Positive environmental impact,” “Reasonable milk alternative,” and “Animal welfare” (Table 3). The adolescents in our study most frequently justified their positive evaluation with the belief that AFM is more environmentally friendly than CM and/or PBMA (*n* = 20). They assumed that less carbon dioxide and methane were emitted in the production of AFM compared to CM production. More generally, they argued that the production of AFM must be more environmentally friendly as no cows are needed to produce it, thus conserving resources. Furthermore, the adolescents in our study justified their positive evaluation of AFM with its similarity to CM, which made AFM a reasonable, desirable alternative to them (*n* = 18). Another justification for the adolescents’ positive evaluation was animal welfare (*n* = 15). They stated that no animals are needed to produce AFM and that, as a result, no animals must suffer compared to conventional milk production. They explained the suffering of dairy cows in detail basing their beliefs on documentaries, images, and video reports on social media (Table 3). In addition, the participants mentioned attributes from the following categories to justify their positive evaluation of AFM: “Innovation” (*n* = 10), “Ethical concerns regarding CM (production)” (*n* = 9), “Facilitating dietary change (*n* = 6), “Curiosity/Interest” (*n* = 5), “Various applications” (*n* = 4), “Anticipated taste” (*n* = 3), “Positive health impact” (*n* = 3), “Naturalness” (*n* = 2), “Price” (*n* = 1), “Efficiency production” (*n* = 1), and “Vegan diet” (*n* = 1; Supplementary Material 5).

**Table 3:** *Top three categories and attributes for the positive evaluation of AFM*

Positive attitudes toward AFM ^a^			
Category	Attributes	Example quote ^b^	Frequency, *n* (%) ^c^
Positive environmental impact	environmentally friendly, (ecologically) sustainable	*“And environmentally friendly I said in connection with my positive evaluation because I personally find the CO*_*2*_*production for […] conventional milk far too high and could be significantly reduced with AFM.”* (Emma, WC) *“I rate AFM as more ecologically sustainable and environmentally friendly because […] on the one hand […] this space for the cows is not needed*,* […] but also the space for the oat or soybean fields is not needed either. I could imagine that no more rainforests would be cut down as a result.”* (Isabella, WC)	20 (20.4)
Reasonable milk alternative	alternative, similar, same	*“AFM is definitely a perfect alternative. It’s almost the same as milk. […] That means that recipes don’t have to be changed*,* and people don’t have to adapt to a new taste.”* (Ella, WC) *“Then I would see AFM […] as an alternative. If you try a recipe with normal milk*,* it usually works better than with an oat milk*,* so I imagine using AFM […] as an alternative.”* (Elias, NWC)	18 (18.4)
Animal welfare	animal-friendly, harmless	*“I said animal-friendly in connection with my positive evaluation of AFM because the cows did not have to spend their lives in cages*,* some of which were far too small.”* (Emma, WC) *„I would say that AFM […] is gentler on the cows, because […] there are also documentaries and pictures in which […] cows have milked twice a day and then their udders […] get sore and so on.”* (Charlotte, WC) *“There is […] no animal that must live under bad conditions […].”* (Elias, NWC)	15 (15.3)

*Notes.*^a^ Positive overall evaluation of AFM (*n* = 22). ^b^ Statements from all adolescents who were willing (WC) or not willing (NWC) to consume AFM to justify their positive evaluations. ^c^ Percentage of all coded segments in the category “Positive attitudes” (*N* = 98)

In addition to the three adolescents who rated AFM negatively overall, the adolescents who evaluated it positively overall also rated AFM negatively in some respects. They justified their negative evaluation of AFM with uncertainties regarding the production process (*n* = 11), the technical and financial costs of production (*n* = 7), and concerns regarding food safety (*n* = 6). Amelia and Lucas explained their uncertainties regarding the production and safety of AFM:

*“I evaluated AFM as rather negative […] because it is unfamiliar to me. Attributes for it could be unfamiliar and new. Even though I have some information*,* it’s still a bit uncertain for me […] how that […] works*,* making AFM.”* (Amelia, willing to consume AFM)

*“One could evaluate AFM rather negatively because the production is unsafe*,* […] so it is still very new and therefore also uncertain […] whether the production of AFM […] works so well.”* (Lucas, willing to consume AFM)

The adolescents also stated in connection with their negative evaluations that they perceived the production of AFM as elaborate, which led to uncertainties regarding food safety:

*“So […] first the milk proteins must be produced in the lab*,* then it also must be done under certain conditions […]*,* so that […] there could be no contamination or anything like that. I imagine that would also be very costly and time-consuming.”* (Lily, willing to consume AFM)

Attributes of the following categories were also mentioned to justify adolescents’ negative evaluation of AFM: “Food technology concerns” (*n* = 5), U“nnatural product” (*n* = 5), “Uncertainties regarding ingredients” (*n* = 4), “Lack of familiarity” (*n* = 3), “Lack of benefit” (*n* = 3), “Negative impact on agriculture” (*n* = 2), “Habit to consume CM” (*n* = 1), and “the Anticipated price” (*n* = 1; Supplementary Material 5).

### Motives for and against PBMA consumption

The adolescents in our study most frequently justified their regular or casual consumption of PBMA with motives in the categories of “Environmental protection,” “Promotion of individual health,” and “Animal welfare” (Table 4). They argued that the negative environmental impact of animal husbandry (e.g., high CO_2_ and methane emissions) could be reduced by consuming plant-based alternatives, thus protecting the environment (*n* = 12). In addition, they frequently justified their consumption by arguing that cow milk consumption harmed their health due to existing intolerances or the milk’s ingredients (*n* = 12). Most of the adolescents in our study stated that sustainability or the protection of the environment and individual health were important to them overall and influenced their consumption decisions. Furthermore, the adolescents in our study explained their consumption of PBMA by stating that the husbandry of dairy cows was morally unacceptable, and that consuming plant-based alternatives protected animal welfare (*n* = 11). The adolescents also cited motives from the following categories for their consumption of PBMA: “Ethical concerns regarding CM (production)” (*n* = 6), “Social influence” (*n* = 6), “Taste” (*n* = 6), “Dietary habits” (*n* = 4), “Social norms” (*n* = 3), “Curiosity” (*n* = 2), “Generational ideals” (*n* = 2), “Variety” (*n* = 2), “Supporting market changes” (*n* = 1), “Availability” (*n* = 1), “Facilitating dietary change” (*n* = 1), and “Familiarity” (*n* = 1; Supplementary Material 6).

**Table 4:** *Top three motives for consuming PBMA*

Category	Example quote ^a^	Frequency, *n* (%) ^b^
Environmental protection	*“The strongest motive is sustainability. […] I think it’s good that by drinking PBMA I’m not forcing animals […] to give me milk […]*,* and I can still live on this planet in 50 years if I keep drinking PBMA and everyone else does the same. That way we wouldn’t need so many cows and so much […] animal agriculture*,* which is a big driver of climate change.”* (Emilia, RC) *“For me*,* the sustainability aspect again counts as a motive. Remember*,* CO*_*2*_*is caused by animal husbandry*,* and I think you can also cook many well-known dishes without animal products.”* (Elias, CC)	12 (17.1)
Promotion of individual health	*“I have developed a slight lactose intolerance over time. Now it’s getting worse and that’s why I’ve switched completely to PBMA.”* (Emilia, RC) *“I think if you drink so much milk now*,* it’s not that healthy. I don’t think nature intended us humans to drink CM. That’s why it’s not so healthy to consume so much CM. And (…) I think an almond drink*,* for example*,* is a healthy alternative.”* (Olivia, CC)	12 (17.1)
Animal welfare	*“Animal welfare weighed most heavily in my decision. […] I don’t see the point of keeping a cow somewhere in a small space if I want milk from it and the calf doesn’t get the milk because of it.“* (Lily, RC) *“One motive would be to avoid the abuse of dairy cows […]. The animals are only used so that the milk yield is as high as possible.”* (Sophie, CC)	11 (15.7)

*Notes.*^a^ Statements from all adolescents who regularly (RC) or casually (CC) consumed PBMA. ^b^ Percentage of all coded segments in the category “Motives for PBMA consumption” (*N* = 70)

The adolescents in our study most frequently justified their non-consumption of PBMA with motives from the categories “Taste and consistency,” “Price,” and “Social influence” (Table 5). The flavor and consistency of certain PBMA were frequently cited by adolescents in our study as a motive for not consuming them (*n* = 11). More specifically, the adolescents explained that they generally disliked the taste of certain PBMA (oat, almond, and soy milk) or that they disliked the flavor and consistency of PBMA in muesli, porridge, or coffee. Another motive for not consuming PBMA was the price compared to CM (*n* = 7). The adolescents in our study stated that they might not consume PBMA because they assumed that they were more expensive than CM. Some adolescents could imagine consuming PBMA regularly if they cost the same or less than CM. Some of the adolescents in our study stated that price determined their consumption decisions due to limited financial resources. In addition to taste, consistency, and price, the adolescents explained that their social surroundings determined their non-consumption of plant-based alternatives (*n* = 6). On the one hand, they described the social attitude toward the consumption of CM as “normal” and explained that this norm encouraged them to not consume plant-based drinks. On the other hand, they argued that the availability of PBMA in their social environment was limited, for example, due to their parents’ consumption behaviors. The adolescents also cited motives from the following categories against their consumption of PBMA: “Social norms” (*n* = 5), “Lack of availability” (*n* = 5), “Convenience” (*n* = 4), “Lack of familiarity” (*n* = 4), “Dietary habits” (*n* = 3), “Negative environmental impact” (*n* = 2), “Uncertainties” (*n* = 2), “Individual ineffectiveness” (*n* = 1), “Ingredients” (*n* = 1), and “Promotion of individual health through CM” (*n* = 1; Supplementary Material 6).

**Table 5:** *Top three motives against consuming PBMA*

Category	Example quote ^a^	Frequency, *n* (%) ^b^
Taste and consistency	*“The taste speaks a little against the consumption of PBMA. […] I find normal milk tastier than PBMA. […] For example*,* oat milk has a strange aftertaste. I find hazelnut milk a bit too sweet. I think almond milk is also a bit too sweet.”* (Jack, RC) *“The taste is an argument against the consumption of PBMA. […] I am also very sure that many people […] are like me and don’t like almond milk*,* for example.”* (Sophia, CC) *“There is nothing that really stops me from consuming PBMA*,* except the taste. The taste is the most decisive reason I don’t yet consume plant-based drinks.”* (Maëlys, NC)	11 (21.2)
Price	*“One motive against PBMA would clearly be the price.”* (Mason, RC) *“Basically*,* I would say that if milk prices become very high*,* which would perhaps be very beneficial for the farmers and for the animals afterward*,* I could imagine switching completely to PBMA.”* (Elias, CC) *“I think PBMAs are even more expensive than milk. For example*,* my mother is a single parent*,* and we can’t spend two euros or more on a liter of “milk” every time […].”* (Isabella, NC)	7 (13.5)
Social influence	*“For me personally*,* the consumption of PBMA depends on the people you are out with and the reaction of others. […] If you’re out with your family or grandparents and you want to explain to them that you only drink oat milk*,* it’s sometimes a bit difficult.”* (Emilia, RC) *“We only have animal milk at home because we live on a former farm and my mother grew up having her own cows and drinking their milk. She sticks with it and doesn’t buy PBMA but continues her tradition.”* (Amelia, NC)	6 (11.5)

*Notes.* ^a^ Statements from all adolescents who regularly (RC), casually (CC), or never (NC) consumed PBMA. ^b^ Percentage of all coded segments in the category “Motives against PBMA consumption” (*N* = 52)

### Motives for and against the willingness to consume AFM

The adolescents in our study most frequently justified their willingness to consume AFM with motives from the categories “Animal welfare,” “Cow milk equivalent,” “Curiosity,” and “Environmental protection” (Table 6). The most frequent justification they gave for their willingness to consume AFM was that it offers a morally acceptable alternative to the consumption of “dairy products,” as no animals must suffer to produce it (*n* = 15). The adolescents in our study also stated the similarity of AFM to CM in terms of taste as a motive for their willingness to consume it (*n* = 13). They explained that a milk alternative that tasted like CM would make it easier for them to forgo CM. They also justified their willingness to consume AFM with their curiosity about new foodstuffs (*n* = 8). In addition, they stated that environmental protection motivated their willingness to consume AFM as its production could reduce greenhouse gas emissions and the amount of land used for dairy farming (*n* = 8). The adolescents also cited motives from the following categories for their willingness to consume AFM: “Ethical concerns regarding CM (production)” (*n* = 6), “Promotion of individual health” (*n* = 5), “Taste” (*n* = 5), “Supporting market changes” (*n* = 4), “Facilitation dietary change” (*n* = 4), “Variety” (*n* = 2), and “Vegan diet” (*n* = 2; Supplementary Material 6).

**Table 6:** *Top three motives for willingness to consume AFM*

Category	Example quote ^a^	Frequency, *n* (%) ^b^
Animal welfare	*“I would say I am simply very committed to animal rights. […] Animal welfare is simply very close to my heart. That’s why I would be willing to consume AFM.”* (Emma, WC) *“I would be willing to consume AFM because then I can drink the milk again with a good feeling […] or eat other dairy products […] because I know that […] no animals are suffering.”* (Maëlys, WC)	15 (20.8)
Cow milk equivalent	*“I […] find it more difficult to give up milk at first. I think if there was an alternative for me that was just like CM*,* then I would say that I would give it a try. If AFM tastes the same as CM*,* then why do I have to drink CM when I have a milk substitute that tastes the same.”* (Maëlys, WC) *“One motive for my willingness to consume AFM is that it should taste the same as milk. If it tastes exactly like milk […] and no animals must suffer for it*,* why not consume AFM? It’s a good alternative.”* (Mathys, WC)	13 (18.1)
Curiosity	*“One motive for consuming AFM is this […] adventurousness*,* […] that you can try out new things. That is very strongly anchored in me […].”* (Samuel, WC) *“I would consume AFM because I find it super interesting. It’s just another opportunity to try out new flavors […].”* (Mia, WC)	8 (11.1)
Environmental protection	*“Environmental protection is a motive for me to consume AFM because I would say that it helps […] that fewer cows must be kept alive. […] This […] reduces methane emissions and therefore also reduces the global warming effect.”* (Charlotte, WC) *“One motive for consuming AFM is actually […] the ecological aspect because it saves space. It’s better for the environment in the long term […]. […] I think AFM […] makes sense above all if there are more and more people in the world in a few years because people also need their space*,* and these cow pastures or fields can then be replaced with living spaces.”* (Isabella, WC)	8 (11.1)

*Notes.*^a^ Statements from all adolescents who were, overall, willing (WC) or not willing (NWC) to consume AFM. ^b^ Percentage of all coded segments in the category “Motives for the willingness to consume AFM” (*N* = 92)

The adolescents in our study most frequently justified their unwillingness to consume AFM with motives from the categories “Food safety concerns,” “Lack of familiarity,” and “Lack of benefit” (Table 7). In general, the adolescents in our study only gave a few motives against their willingness to consume AFM. The most common justification was their lack of familiarity with and knowledge about AFM and its production (*n* = 3), which raised concerns about food safety (*n* = 3). The adolescents in our study also justified their unwillingness to consume AFM by indicating that they did not see the need for another milk alternative (*n* = 2). In addition, the adolescents cited motives from the following categories against their willingness to consume AFM: “Ethical concerns AFM (production)” (*n* = 2), “Dietary habits” (*n* = 2), “Lack of technical understanding” (*n* = 2), “Negative impact on agriculture” (*n* = 2), “Anticipated taste” (*n* = 1), “Anticipated price” (*n* = 1), “Individual health disadvantages” (*n* = 1), and “Individual ineffectiveness” (*n* = 1; Supplementary Material 6).

**Table 7:** *Top three motives against willingness to consume AFM*

Category	Example quote ^a^	Frequency, *n* (%) ^b^
Food safety concerns	*“One argument against the consumption of AFM is that it is very time-consuming and again this risk. […] I have the feeling that AFM could be risky because something could go wrong when copying the DNA […]*,* the cells then mutate and AFM […] then becomes […] unhealthy or even dangerous for the body.”* (Jack, WC) *“What speaks against the consumption of AFM for me is that you don’t know exactly what AFM […] consists of*,* how AFM […] is produced*,* and whether AFM […] is perhaps dangerous for your health after all.”* (Mathys, WC)	3 (15)
Lack of familiarity	*“One motive against the consumption of AFM is that the production itself […] is not yet so well-known […] and one does not know exactly what else is in it.”* (Lucas, WC) *“The production of AFM […] seems quite abstract to me and I think that makes it rather uninteresting for me*,* which is why I’m rather unwilling to consume AFM.”* (Elias, NWC)	3 (15)
Lack of benefit	*“What speaks against the consumption of AFM for me is that I don’t find it necessary.”* (Oliver, WC) *“I would perhaps […] not be prepared to continue consuming AFM after trying it because of the production process because the production is very time-consuming*,* and I don’t know whether people need it […]. AFM is an alternative and if you like it*,* it’s good*,* but I don’t know if it’s necessary to go through such a complex production process […] just to have […] a milk alternative that is like milk*,* although we have many other alternatives to milk.”* (Emmy, WC)	2 (10)

*Notes.*^a^ Statements from all adolescents who were overall willing (WC) or not willing (NWC) to consume AFM. ^b^ Percentage of all coded segments in the category “Motives for the willingness to consume AFM” (*N* = 20)

## Discussion

### Attitudes toward milk alternatives

#### Perceptions of PBMA (compared to CM)

While studies with adults have shown that CM products are perceived as healthy due to their protein and calcium content [50, 57, 88], for example, our results suggest that young people (such as those in Generation Z) are becoming more aware of the effects of CM consumption on individual, animal, and environmental health. This could lead adolescents to question the positive product image of CM. Their belief that PBMAs are a healthy alternative to CM could also be a result of the increasingly critical discussion of the consumption of CM in conventional and social media in recent years—especially the effects of consuming animal foods on health, animal welfare, and the environment [89, 90].

Regarding their perception of PBMA as environmentally friendly, participants mentioned that they were convinced that only some milk alternatives are more environmentally friendly than CM. For example, they classified oat milk as environmentally friendly and soy milk as environmentally unfriendly because they believed that oats can be grown regionally, whereas soy can only be grown in tropical areas, meaning that rainforests must be destroyed to obtain land for soy milk production. Although the deforestation of rainforests is linked to soy production, the soy produced thereby is mainly used for animal feed [91, 92]. This misconception led to a negative perception of soy milk among our participants.

Our results suggest that beliefs about the health, animal welfare, and environmental impacts of consuming CM and PBMA play a major role in whether PBMA are evaluated as positive or negative. The price of PBMA, feelings about their taste and consistency, and misconceptions about their impact on the environment and health can also cause consumers to evaluate them negatively. In this context, Martinez-Padilla et al. [60] found that perceiving PBMA as healthy, tasty, or nutritionally equivalent to CM is positively related to the consumption of PBMA. This suggests that beliefs about the positive effects of consuming PBMA on individual and environmental health are crucial for promoting positive attitudes toward and the consumption of PBMA [60].

#### Perceptions of AFM (compared to CM)

Our participants justified their positive attitudes toward AFM with their negative beliefs and feelings about dairy farming. They thoroughly explained how dairy cows suffer for CM production, basing their beliefs on documentaries, images, and video reports that they saw on social media. Similarly, German adults perceive animal-free cheese as more ethical and environmentally friendly than conventional cheese [31]. Animal welfare appears to be a key argument for the positive perception of animal-free dairy [32]. In line with the results of Broad et al. [32], our participants had concerns about conventional animal husbandry that would not exist with animal-free dairy products.

Contrastingly, uncertainties about the ingredients, production process, and food safety of AFM, which were driven by a lack of familiarity with AFM and its production, fostered negative attitudes toward AFM among our participants. However, Zollman Thomas and Dillard [63] found that despite perceiving animal-free cheese as artificial compared to plant-based and conventional cheese, their participants were willing to try it. This ambivalence was also observed in our study: Participants, for the most part, were willing to consume AFM, despite their uncertainties about its ingredients and production. Our results suggest that beliefs about the advantages and safety of AFM are weighted differently in the decision to consume AFM and that uncertainties do not necessarily negatively influence the willingness to consume novel foods. Nevertheless, Broad et al. [32] emphasized that assuaging uncertainties and concerns is crucial for fostering positive attitudes toward animal-free dairy and the willingness to consume it, as their participants were undecided on the consumption of animal-free dairy since they lacked information and had uncertainties about these products. This underlines the public’s need for information on the ingredients, production, and safety of animal-free dairy products, especially the safety of consuming animal-free dairy for individual health [32].

#### Suggestions for education and marketing

As the consumption of milk and dairy is still an integral part of many dietary guidelines and dietary habits in European countries [93], education and marketing should be aimed at promoting positive attitudes toward the consumption of milk and dairy alternatives at an early age, as these attitudes could influence consumer behavior [53, 94]. However, previous research has demonstrated a discrepancy between consumers’ attitudes and consumption behavior [95, 96].

In German biology classes, young people learn that the German Nutrition Society recommends the daily consumption of milk and dairy products because they contain easily available protein and essential micronutrients such as calcium, vitamin B12, and iodine. However, the German Nutrition Society also defines a healthy and environmentally friendly diet as one comprising about three-quarters plant-based and one-quarter animal-based products [14, 97]. As our current diets consist mainly of animal-based products [4, 93], young people should be informed about the nutritional value of CM and PBMAs, as well as their environmental impact, to prevent misunderstandings and enable young people to make informed dietary choices.

Marketing strategies should be focused on the health benefits of PBMA compared to CM (e.g., being lactose-free) while communicating their unique nutrient profiles [27]. In addition, emphasizing the unique selling points of PBMA (e.g., the protein content of soy milk) could also help to prevent misunderstandings. For AFM, marketing strategies should be focused on promoting animal and environmental welfare, as well as the similarities between AFM and CM, to appeal to current milk consumers [32]. Kossmann et al. [47] found that the perceived advantages of animal-free cheese, such as its sustainability, have a positive effect on consumers’ willingness to buy it. It has already been shown that attitudes toward novel foods are crucial for market introduction and that they can be improved by providing information about the benefits of these foods [98]. In addition, uncertainties and concerns about AFM and its production should be minimized by providing information about its production process and safety for individual health [32, 63]. However, AFM is not yet approved for sale on the European market, which may lead to uncertainties about the safety of AFM among potential consumers. Such uncertainties could negatively impact consumers’ willingness to buy dairy produced via precision fermentation [47]. Therefore, the approval of AFM for sale on the European market is a crucial factor in reducing uncertainties about and increasing consumer acceptance of AFM.

### Motives for and against the consumption of milk alternatives

#### Motives for consuming PBMA

The decision to consume or not consume PBMA among our participants was based on a complex interplay of individual (e.g., the motivation to protect individual health, animals, and the environment) and external (e.g., family and society) factors. Our participants questioned the sustainability and necessity of current livestock farming levels—especially given the ongoing climate change they have grown up with [99]. In addition, existing intolerances, such as lactose intolerance [22, 100], and their desire for a healthier diet motivated participants to reduce their consumption of products containing lactose, fat, and sugar. Furthermore, Giacalone et al. [72] noted that not all plant-based alternatives are perceived as equally environmentally friendly and healthy. In our study, for instance, participants considered oat milk an environmentally friendly alternative to CM, as oats can be grown regionally in Germany; similarly, almond milk was considered a healthy alternative to CM since it has lower calorie and fat content.

Animal welfare was also a crucial motive to consume PBMA among our participants and was linked to the moral stance against the abuse of animals for food production. Adults also consider animal welfare to be one of the most important considerations when buying dairy products or plant-based dairy alternatives [44, 101]. The growing awareness of health, animal welfare, and the environment is also reflected in the dynamic development of young people’s dietary habits in Germany. Young people (aged 15 to 30) are increasingly avoiding the consumption of animal products for climate and animal welfare [62, 102].

#### Motives against consuming PBMA

As our results show, the sensory characteristics of PBMA are a major barrier to their consumption [59, 72, 103, 104]. American children and adolescents (aged 8 to 16), for example, rated lactose-free milk as better, in general, than soy milk since lactose-free milk had better sensory properties, such as taste and mouthfeel [61]. In addition, the price of PBMA compared to that of CM—perceived or real—was a motive against the consumption of PBMA. This motive is important for younger people, as they have limited incomes and depend on their family’s shopping and dietary habits [99] and since one in five young people in Germany grows up in a low-income household [105]. In addition to individual factors, environmental factors affect sustainable food consumption [106]. We found that social influence and social norms related to the consumption of CM are also barriers to the consumption of PBMA, as CM consumption is often considered “normal” and an integral dietary habit [71, 97, 107].

#### Motives for the willingness to consume AFM

The willingness of our participants to consume AFM depended mainly on individual factors, such as a concern for animal welfare. Animal welfare was a key motive to consume milk alternatives (AFM and PBMA) among our participants, as they questioned the necessity and conditions of contemporary livestock farming. The importance of animal welfare for younger people when making food choices is reflected in Generation Z’s increasing commitment to animal welfare [99, 108]. Similarly, Zollman Thomas and Dillard [63] showed that promoting animal welfare or reducing animal suffering in livestock farming is the strongest motivation for adults’ consumption of animal-free dairy (e.g., cheese or milk). The feeling of unease about livestock farming drives the need to protect animal welfare and thus avoid consuming animal products. This unease can be enhanced by presenting information about or showing pictures of the conditions of dairy farms (“animal welfare framing”), which could encourage consumers to consume animal-free dairy [63].

#### Motives against the willingness to consume AFM

First, most of our participants were very interested in trying AFM and gave few motives against consuming it. Regarding their justifications for their negative attitudes toward AFM, uncertainties about its safety for human health were mentioned. Zollman Thomas and Dillard [63] observed that uncertainties about dairy products produced with precision fermentation hinder their acceptance. The European Food Safety Authority’s (EFSA) approval of AFM for sale on the European market could help to reduce uncertainties about AFM. In addition, all companies and marketing campaigns should use a consistent description of the production process of AFM to minimize uncertainties and facilitate understanding of “animal-free dairy” as a product category [63].

#### Suggestions for marketing and policy

Young people’s growing awareness of health, animal welfare, and the environment, as well as their doubts about our current food system [99, 102], could be a good starting point for developing marketing campaigns for CM alternatives. Based on our results, marketing campaigns should be aimed at emphasizing the health, ethical, and environmental benefits of consuming PBMA instead of CM and directly addressing misconceptions (e.g., rainforest deforestation to produce soya milk). Since our results show a discrepancy between young people’s attitudes toward and consumption of PBMA (attitude–behavior gap), we should consider how existing barriers can be overcome.

The barrier of flavor and consistency may result from the perception of CM as “nice,” while PBMA do not meet sensory expectations in comparison [59, 69, 71]. This barrier among “CM lovers” may be overcome by producing PBMA with sensory properties similar to those of CM. One example of this is Alpro’s “Not Milk,” which is available on the German market and is advertised with the slogan “Not milk, but it tastes like milk” [109]. Advertising that highlights milk alternatives’ similarity to CM could be particularly important for people who have not yet been able to eliminate CM since they prefer its flavor. Another barrier to the consumption of PBMA is cost. PBMA are currently taxed at 19% in Germany, while CM is taxed at 7% since it is considered a staple food there. Equalizing taxes on CM and PBMA is one way to lower the price barrier [110]. Furthermore, the potential for the social environment to promote the consumption of PBMAs should be considered since family and friends influence adolescents’ food choices [111]. Adolescents also influence each other’s diets and food choices (e.g., via social media) [112]. This could be used to actively drive the normalization of PBMA consumption.

The approval of animal-free dairy products in Europe could allay safety concerns. Hence, communication should be focused on the advantages of AFM, as previous research has shown that the perceived benefits of animal-free cheese have a positive effect on the willingness to buy it [47]. In addition, previous studies have suggested that companies should make it clear when AFM has not been produced using genetically modified organisms (GMOs), as associating AFM with GMOs could have a negative impact on risk perception and willingness to consume [49, 63, 113]. Open, transparent communication about the new product category of animal-free dairy products could also help to increase familiarity, which is crucial for the willingness to consume novel foods [98]. Furthermore, attention should be paid to the placement of AFM in the supermarket when it is introduced; for example, placing AFM close to CM could promote familiarity with the product [32]. In summary, AFM could become a suitable milk alternative among younger people [31]. Most of our participants already perceived AFM as a reasonable milk alternative. Whether they will integrate AFM into their diets will mainly depend on their food choice motives, such as price and taste, and the assuaging of uncertainties about AFM in marketing campaigns.

### Limitations

Since we used a qualitative interview approach and had specific inclusion criteria for our sample, we did not aim for a sample that was representative of young people in Germany. Therefore, based on our results, we could not make generalized statements about young people’s attitudes toward milk alternatives and their motives for or against consuming them [82]. Future studies could examine attitudes toward milk alternatives and motives for or against consuming them among adults or young people following another eating culture. Another idea for further research would be to examine the attitudes of young people from the United States, where AFM is already available on the market. Moreover, all our participants were engaged in higher education (upper secondary level), which may have affected our results since previous studies have shown that people with high levels of education are more likely to adopt sustainable dietary habits [114, 115].

In addition, only the most frequently mentioned product attributes and motives for consuming milk alternatives were presented and discussed in detail. Less frequently mentioned categories were only mentioned, but not further explained with anchor quotations, which represents a methodological limitation. It is important to consider this methodological limitation when interpreting the results.

Self-selection bias was another major limitation of this study. Participation in the study was voluntary, which could have biased the sample since only students interested in sustainable food choices volunteered to participate. In this context, the results may also have been distorted by social desirability–particularly in responses about the willingness to consume AFM. Of the 25 participants, 23 were willing to consume AFM, which could have been as a result of an improvement in self-presentation [116]. Additionally, food choices are not only determined by cognitive factors such as willingness to consume; they are also determined by personal (e.g., psychological components and habits), external (e.g., the social environment), and sociocultural (e.g., culture, economic variables) factors [117]. These factors should be considered when interpreting our results since there may have been differences between participants’ statements about their consumption of milk alternatives and their actual behavior.

Furthermore, 24 of the 25 participants stated that they have the right to co-determination when making purchases. Participants’ opportunities, cultural background, and level of education should be considered when interpreting our results since our results do not apply to adolescents from other educational backgrounds, younger people, or people from different cultural backgrounds. Differences in perceptions of PBMA and AFM can occur due to traditions and cultures [31, 57]. In addition, adolescents are largely financially dependent on their parents and do not have to make their own purchases. Therefore, young people’s attitudes and consumer behavior could change after they leave their parents’ homes. Particularly during the transition from school to university, new lifestyles often develop, and existing habits are reconsidered [118].

Labeling milk produced via precision fermentation as “AFM” was another methodological limitation. Zollman Thomas and Dillard [63] demonstrated that using different names for dairy products produced with precision fermentation has different effects on potential consumers. Following Zollman Thomas and Dillard’s [63] recommendations, we used the term “AFM” because it is simple and clearly differentiates AFM from CM. Nevertheless, the use of the English term “AFM” may have confused participants during the interviews, with some mistakenly thinking that it referred to PBMAs.

## Conclusions

Our results showed that the adolescents in our study generally had positive attitudes toward PBMA and AFM. However, uncertainties about the production of AFM and its safety for human health diminished their positive attitudes. In addition, our results indicated that adolescents’ decisions to consume PBMA were driven by environmental protection and health promotion motives. The main motives to consume AFM were animal welfare and the equivalency of AFM to CM (e.g., in taste and consistency). Despite the perceived benefits of PBMA and AFM, their perceived sensory properties, costs, and production uncertainties represented barriers to their consumption.

Based on our results, we derived implications for further research, education, and the food industry. Future studies could comparatively analyze young people’s and adults’ attitudes toward CM and dairy alternatives, as well as their motives for or against consuming these products. Another idea for further research would be to compare the attitudes and motives of young people in a country with a strong tradition of consuming dairy products, such as Germany, with those of young people in a country with a weaker tradition of consuming dairy products. Furthermore, we recommend that as the range of alternative products grows, the environmental impact and nutritional values of traditional foods and alternative products be discussed in more depth in the context of Education for Sustainable Development to reduce misconceptions about milk and milk alternatives and promote informed food choices. For the food industry, our results suggest that product communication for PBMA should be focused on the health benefits of such products and transparency in the unique nutritional profiles of various milk alternatives. For AFM, the positive attitudes of our sample provide a good starting point for market introduction. That is, during its introduction, its similarities to traditional milk, as well as its environmental benefits, should be emphasized. To overcome safety concerns about AFM, the EFSA’s approval of AFM for sale on the European market is essential.

## Electronic supplementary material

Below is the link to the electronic supplementary material.

Supplementary Material 1

Supplementary Material 2

Supplementary Material 3

Supplementary Material 4

Supplementary Material 5

Supplementary Material 6

## Data Availability

The datasets used and/or analyzed during the current study are available from the corresponding author upon reasonable request.
