# Supplementary material for: Attitudes toward milk alternatives and motives for (non-)consumption: an interview study with adolescents from Germany
Source: BMC Nutr. 2025 May 26;11:101. doi: 10.1186/s40795-025-01072-8 (PMC12105291; doi:10.1186/s40795-025-01072-8)
Supplement: Supplementary file 1 — Supplementary Material 1 [file 40795_2025_1072_MOESM1_ESM.docx]

Osnabrück University

Department of Biology/Chemistry

Biology Didactics

| Semi-structured interview guide* |
| --- |
| Milk Alternatives - Attitudes, Motives and Consumption Behavior/Willingness to Consume among Adolescents in Germany |

An explorative interview study with adolescents from secondary schools in the city and region of Osnabrück

*Note*. *Only the parts/questions of the interview relevant to the publication are presented.

| **Card:** 1 | **Section:** 1 - Greeting and small talk | **Intention / Research Interest:** To allow adolescents to arrive in the situation | |
| --- | --- | --- | --- |
| **Preparations**   - Place: classroom or other room in the school - Position tables and chairs - Provide drinks (water, Lemonades) and 2 glasses. - Provide cookies - Get recording device ready (2x plus batteries plus iPhone) (test recording function and prepare recording) - Two notepads, two pens, an iPad with an interview guide (as A5 index cards), a questionnaire, and working material for the respondents on the table. - Have oat drink packaging ready   **Procedure**   - Welcome - Sit down - Short small talk: - "How are you?" - "Do you feel like some water or a Lemonade?"; If so, "I'll pour you some already." - "What class did you just have?" "What interesting things did you cover in class today?" - "Have you ever participated in an interview?" - Clarify schedule:   - "Do you have any subsequent deadlines that I need to consider? The interview will take about one school hour. We could finish earlier, but it won't take longer than that." | | | |
| **Card:** 2 | **Section:** 2 - Introductions and information about the interview | | **Intention / Research Interest:**  Clarification of the general conditions and the procedure of the interview |
| **Procedure:**  (Switch on recording device 1 (iPad))   1. **Introduction**  - "Before the interview starts, I'd like to give you some information about the interview process and how I'm going to use the information. I'll start with me first, though."  1. **Introduction of the Interviewer**  - "My name is Lena and I work in biology didactics at the University of Osnabrück. As part of my doctoral thesis, I'm working on the topic of sustainable nutrition, and that's exactly why I'd like to talk to you today."  1. **Information on the subject and the tasks of the interview**  - Topic: Milk alternatives - Themes: Associations and consumption (reasons). - Note to adolescents: tell own thoughts and ideas, no wrong answers, ask follow-up questions.   - "More specifically, today we're going to talk about the topic of "milk alternatives." In our conversation today, I'm not interested in your knowledge on the subject of "milk alternatives". Rather, I'm interested in your ideas and thoughts on selected milk alternatives and also whether you already drink/would drink milk alternatives and why."   - "When I ask you my questions in a moment, it's important that you just tell me everything that pops into your head. There are no wrong or bad answers. I just want to know what you imagine, what you think, and why you think that when you ask each question about milk alternatives." Your ideas and thoughts about specific biology class topics help us improve our class materials and biology classes. If you have a question or ever don't understand me, you can always ask follow-up questions. Do you have a question so far?"  1. **Procedere, legal, technology**  - "Unfortunately, I can't remember everything you say, so I brought a recording device. I use it to record our conversation so that I can listen to it again later. The recording will be typed up afterwards and the recording will be deleted, since I will only continue to work with the written document for my work." - "Everything you tell me today and what we talk about will remain between us. No one will know what you said in our conversation. Neither your school administration nor your biology teacher nor your classmates will know what you said. Your name will not appear anywhere in my work. Therefore, you don't have to worry about saying something wrong." - "At any time during the call, you can communicate if you need a break or want to end the call immediately. You are welcome to put your cell phone on the table. However, it would be good if you put it on silent. If someone calls you during the interview, you can answer and/or leave the room for a short phone call. I'm just going to turn on the recorder now, but I'm not going to record anything yet. Because I'll have to ask you again in a minute if I can record our conversation so I can have your answer on tape." - "After the interview, I would like to fill out a short questionnaire with you. The questionnaire contains general questions about yourself and topics that will be addressed in the interview."  1. **Notes**    - "In between, I'll be taking notes every now and then, so I've got a pen and pad ready. On the cards here, I've written my questions that I want to ask all the students. So, I don't forget any questions, the cards are here. Every now and then I put a card aside, but don't let that confuse you. 2. **Questions, consent**     - "Do you have any questions about the interview?" (Switch on recording device 2 (recorder))    - "If you have no more questions, I will start recording now. Are you okay with that?" | | | |

| **Card: 5** | **Section:** 4 - Main Phase 1, Block 2  **Topic:** Attitudes toward plant-based milk alternatives | | | **Intention / Research Interest:**  What are adolescents attitudes toward plant-based milk alternatives? | |
| --- | --- | --- | --- | --- | --- |
| **Guiding question/narrative prompt:**  "How would you evaluate plant-based milk alternatives now? Positive or negative?" (1)  "You have rated plant-based milk alternatives as positive/negative. Now try to explain to me with three adjectives (how-words) why you rated plant-based milk alternatives as positive/negative." (3) | | | | | |
| **Alternative wording(s):**  "Would you now rate plant-based milk alternatives as positive or negative?" (2)  "Can you try to explain to me with three adjectives (like-words) why you rated plant-based milk alternatives as positive/negative." (3) | | | | | |
| **Content aspects:**   - Capture attitudes toward plant drinks by (1) rating the product " plant-based milk alternatives " as an overall evaluation of the object as "positive" or "negative" and (2) justifying the rating with three adjectives. - Recording of positively and negatively connotated adjectives (affective, cognitive, conative) associated with the product "plant-based milk alternatives". (2) - Interviewer takes notes on the evaluation and notes the adjectives | | **Further questions / notes:**   - After (1): If subjects are undecided: "More positive or more negative?" - To (2): Give the subjects the hint that the adjectives may be written down. - after (2): ask for an explanation and evaluation of the respective adjectives, for example: "Can you explain X (adjective) to me again in more detail?"; "Can you explain to me again in more detail how X is related to your positive/negative evaluation?" - after (2): "Do you also associate positive/negative adjectives with plant-based milk alternatives?" (contrary to the individual evaluation of the respondent) | **Intervention / Other:**  / | | **Possible answers:**   - Regarding (1): Evaluation is mixed, but rather positive (Kempen et al., 2016). - to (2): Adjectives of affective, cognitive, and conative dimensions in domains according to Markinova et al. (2015). - Health (Haas et al., 2019). - Mood regarding consumption - Convenience in terms of production and consumption, e.g., easy (Markinova et al., 2015), accessible (Racey et al., 2017). - Environmental & ethical concerns - Naturalness, e.g., unnatural (McCarthy et al., 2017), pollution-free (Markinova et al., 2015). - sensory properties - Price - Habits, e.g. vegan (Markinova et al., 2015). |

| **Card: 6** | **Section: 5** - Main Phase 1, Block 3  **Topic:** Consumption of plant-based milk alternatives - consumer behavior and motives | | | **Intention / Research Interest:**  What is the consumption behavior of adolescents of plant-based milk alternatives?  What are adolescents motives for the (non-)consumption of plant-based milk alternatives? | |
| --- | --- | --- | --- | --- | --- |
| **Guiding Question/Narrative Prompt:**  "Now we've talked about your evaluation of plant-based milk alternatives. Do you already drink plant-based milk alternatives?" (1)   - If so, "What plant-based milk alternatives do you drink? If you think about a normal school week, how often do you drink plant-based milk alternatives?"   "Now I'm interested in your motives for (not) consuming plant-based milk alternatives. Why do you (not) drink plant-based milk alternatives (as an alternative to cow's milk)?" (2) | | | | | |
| **Alternative wording(s):**  "Now we've talked about your evaluation of plant-based milk alternatives. Do you already drink plant drinks?" (1)   - "Can you describe for me which and how often you drink plant-based milk alternatives in a normal school week?"   "You said you (don't) drink plant-based milk alternatives on (X basis). Can you explain to me what your motives are for (not) drinking plant-based milk alternatives?" (2) | | | | | |
| **Content aspects:**   - Self-report on current consumption patterns of plant-based milk alternatives (type of consumption, frequency of consumption) (1). - Explaining consumer behavior by citing motives (2). | | **Further questions / notes:**   - (1) more specific questions on consumer behavior adapted to the respondents' answers, e.g.   - "What plant-based milk alternatives do you consume most often during a normal school week?"   - "On how many days and when/on what occasion do you drink plant-based milk alternatives?"   - "Do you drink plant-based milk alternatives every day?"   - "On the X days you plant-based milk alternatives, how often and why do you drink plant-based milk alternatives?"   - "Do you also drink cow's milk during a normal school week, or do you replace cow's milk entirely with plant-based milk alternatives?"   - "Reflecting on your consumption of plant-based milk alternatives, would you classify it as regular or occasional based on what you've reported?" - to (2): Interviewer notes down the reasons for/against the consumption of plant-based milk alternatives on a ready-made list (M1). - on (2) ask for explanations of the motives, e.g., "Can you explain the motive ... to me again in more detail?" - After (2) demand to prioritize motives, such as:   - "What is the most important motive for/against consuming plant-based milk alternatives for you?"   - "If you had to choose, what would be your most important motive for/against consuming plant-based milk alternatives?" - after (2) "Are there also motives for you that speak against/for the consumption of plant-based milk alternatives?" (contrary to the individual consumption behavior of the test person). | **Intervention / Other:**   - for (2) definition of "motives", if applicable - For (2): Prepared material (M1) for noting the motives for consumer behavior. | | **Possible answers:**   - To (1): Consumption behavior depends on diet and individual preferences & motives, but in principle there is a willingness to try plant-based drinks. - (1): Oat drink and soy drink as most popular plant drinks (German Federal Statistical Office, 2022) - (2): food choice motives (Markinova et al., 2015; Pieniak et al., 2013).   - Sensory perception, e.g., taste, smell (Hamilton, 2006; McCarthy et al., 2017, Pritulska et al., 2016; Racey et al., 2017).   - Price (Hamilton, 2006; Kumar, & Babu, 2014; Pichler, 2009; Pritulska et al., 2016; Racey et al., 2017).   - Ingredients (naturalness) (McCarthy et al., 2017, Pichler, 2009).   - Health (Kerschke-Risch, 2014; McCarthy et al., 2017; Pritulska et al., 2016).   - Amenity, convenience (Racey et al., 2017).   - Familiarity (Racey et al., 2017).   - Society & culture, e.g., co-determination in purchasing (Van der Bergh & Pallini, 2018).   - Environmental impacts & ethical concerns, e.g.   - Animal Welfare (Leitzmann, & Keller, 2013; McCarthy et al., 2017; Prituslka et al., 2016),   - Environmental Protection (Hamilton, 2006, Pritulska et al., 2016, Topić, & Mitchell, 2019) |

| **Card: 9** | **Section: 7** - Main Phase 2, Block 2  **Subject area:** Attitudes towards "animal-free milk" | | | **Intention / Research Interest:**  What are adolescents’ attitudes towards "animal-free milk"? | |
| --- | --- | --- | --- | --- | --- |
| **Guiding Question/Narrative Prompt:**  "You have already told me a few things about your conceptions of "animal-free milk". Now I am interested in how you would evaluate "animal-free milk". But first I will give you some information about what scientists understand by "animal-free milk" and how "animal-free milk" is produced. For this purpose, I have brought you a short informational text. Please read it carefully and then explain to me briefly and in your own words what animal-free milk is and how it is produced. (1)  *Intervention*  "Now I have given you information about animal-free milk. How would you rate animal-free milk: positively or negatively?" (2)  "You have rated "animal-free milk" as positive/negative. Now try to explain to me with three adjectives (like-words) why you rated "animal-free milk" as positive/negative." (3) | | | | | |
| **Alternative wording(s):**  "Now would you rate animal-free milk as positive or negative?" (2)  "Can you try to explain to me with three adjectives (like-words) why you rated "animal-free milk" as positive/negative." (3) | | | | | |
| **Content aspects:**   - to (1): create theoretical basis for the evaluation of the product "animal-free milk"; parallel framing of the test person by information text with focus on technical information. - Recording of attitudes towards "animal-free milk" by (2) the evaluation of the product "animal-free milk" as an overall evaluation of the object with "positive" or “negative" with (3) a justification of the evaluation with three adjectives - Recording of positively and negatively connotated adjectives (affective, cognitive, conative) associated with the "animal-free milk" product. (3) - Interviewer takes notes on the evaluation and notes the adjectives | | **Further questions / notes:**   - after (1): "Do you have any questions about the information text?"/.   "Are there things still unclear to you?"   - After (1): "Now try to explain to me in your own words what "animal-free milk" is and how it is made." - After (2): If subjects are undecided: "More positive or more negative?" - For (3): The subjects are asked to name at least 3 adjectives. - after (3): ask for an explanation and evaluation for the respective adjectives, such as "Can you explain X (adjective) to me again in more detail?"; "Can you explain to me again in more detail how X is related to your positive/negative evaluation?" - after (3): "Do you also associate positive/negative adjectives with "animal-free milk"?" (contrary to the respondent's own evaluation) | **Intervention / Other:**   - Information text on "animal-free milk" (Material M2) and highlighter for marking (if needed) is provided. | | **Possible answers:**   - to (1): animal-free milk alternative (equivalent to cow's milk); water and cow's milk proteins as main ingredients, synthesis of cow's milk proteins by microorganisms - on (2): evaluation ambivalent (Zollman Thomas & Dillard, 2022).   - positive assessment: resource-efficient, modern   - negative rating: artificial - For (3): naming at least three adjectives from affective, cognitive, conative dimensions in the following domains (Zollman Thomas & Dillard, 2022):   - Health, e.g. (un)healthy, low/nutrient-rich, low-calorie, low-fat   - Mood regarding consumption, e.g., skeptical, uncertain   - Environment, e.g., environmentally friendly/harmful, sustainable   - Naturalness, e.g., natural/unnatural |

| **Card: 10** | **Section: 8** - Main Phase 1, Block 3  **Topic:** Consumption willingness of "animal-free milk" - willingness to consume and motives | | | **Intention / Research Interest:**  What is adolescents’ willingness to consume "animal-free milk"?  What are adolescents’ motives for the (non-)willingness to consume “animal-free milk”? | |
| --- | --- | --- | --- | --- | --- |
| **Guiding Question/Narrative Prompt:**  "Now we've talked about your evaluation of animal-free milk. Now imagine that animal-free milk was available in every supermarket and restaurant. Would you be willing to drink animal-free milk?" (1)  "Now I am interested in your motives for/against your willingness to consume "animal-free milk". Why would you (not) consume "animal-free milk"?" (2) | | | | | |
| **Alternative wording(s):**  "Now we've talked about your ideas on animal-free milk. Would you be willing to drink animal-free milk?" (1)  "You said that you would (not) be willing to drink animal-free milk. Can you explain to me what your motives are for (not) drinking animal-free milk?" (2) | | | | | |
| **Content aspects:**   - Self-report on the willingness to consume "animal-free milk" (1) - Explanation of the willingness to consume by citing motives (2) | | **Further questions/notes:**   - to (1): Level of willingness to try "animal-free milk”. - to (2): The interviewer notes down the reasons for/against the willingness to drink/try "animal-free milk" on a ready-made list (M3). - To (2): ask for explanations of the motives, e.g., "Can you explain the motive ... to me again in more detail?" - After (2): demand to prioritize motives, such as:   - "What is the most important motive for you for/against consuming animal-free milk?"   - "If you had to choose, what would be the most important motive for you for/against consuming animal-free milk?" - after (2): "Are there also motives for you that speak against/for the consumption of "animal-free milk"?" (contrary to the willingness of the test person to consume) | **Intervention / Other:**   - for (2) definition of "motives", if applicable - For (2): Prepared material (Material M3) to note down the motives for the willingness to consume. | | **Possible answers:**   - to (1): heterogeneous results regarding the willingness to consume "animal-free milk", general willingness to try "animal-free milk". - To (2): food choice motives (Markinova et al., 2015; Pieniak et al., 2013), e.g., price, naturalness (unnatural, synthetic), health (low in nutrients), society & culture (environmental impact, animal welfare). |

| **Card: 11** | **Section:** End | **Intention / Research Interest:**  Conclusion of the interview, clarify questions.  Supplements |
| --- | --- | --- |
| **Procedere:**   - Fade out/Reconciliation questionnaire - "Now we've talked about some things. Is there anything else you'd like to add, or do you have any questions for me?" - "Did you have any difficulties understanding during the interview? Did you find certain things particularly difficult or distracting? You're allowed to be honest with me! Your feedback might help me do something better during the next interview." - End recording - Fill out the questionnaire together (Appendix A - Part 1)   "Now I have a few final questions about your attitudes and willingness to consume milk alternatives, as well as about yourself."   - Conclusion   "Perfect, that's all the information I need. Thank you so much for participating and I hope you had a little fun. I just have one last request for you. Since I will be conducting the interview with other students at your school, it is important that you do not share any information about the topics we talked about with your classmates. You can probably imagine that it will otherwise distort the results of my study if your classmates already know the exact questions before the interview or know that the topic is "milk alternatives".   - Say thank you, hand over the voucher, have confirmation signed, and say goodbye. - Complete your own reflection questions from the short questionnaire (M4). | | |

**Literature**

Adamczyk, D., Jaworska, D., Affeltowicz, D., & Maison, D. (2022). Plant-Based Dairy Alternatives: Consumers' Perceptions, Motivations, and Barriers - Results from a Qualitative Study in Poland, Germany, and France. *Nutrients, 14,* 2171. https://doi.org/10.3390/nu14102171

Bebié, A. (1978). Buyer behavior - a psychological-sociological account. In: Buyer behavior and marketing decisions. Gabler Verlag. https://doi.org/10.1007/978-3-663-06888-4_3

Drenowski, A. (2021). Plant-based milk alternatives in the USDA Branded Food Products Database would benefit from nutrient density standards. *Nature Food, 2,* 567-569. https://doi.org/10.1038/s43016-021-00334-5

Haas, R., Schnepps, A., Pichler, A., % Meixner, O. (2019). Cow Milk versus Plant-Based Milk Substitutes: A Comparison of Product Image and Motivational Structure of Consumption. *Sustainability, 11,* 5046. https://doi.org/10.3390/su11185046

Hamilton, M. (2006). Eating Death. *Food, Culture & Society, 9* (2), 155-177.

Kempen, E., Kasambala, J., Christie, L., Symington, E., Jooste, L-, Van Eeden, T. (2016). Expectancy-value theory contributes to understanding consumer attitudes toward cow's milk alternatives and variants*. International Journal of Consumer Studies, 41*, 245-252. https://doi.org/10.1111/ijcs.12331

Kerschke-Risch, P. (2014). Vegan diet. Motives, approach and duration - Initial results of a quantitative sociological study. *Ernährungs Umschau International, 6*, 98-103.

Kumar, A., & Babu, S. (2014). Factors Influencing Consumer Buying Behavior with Special References to Dairy Products in Pondicherry State. *International Monthly Refereed Journal of Research in Management & Technology, 3*, 65-73.

Kurajdova, K., & Taborecka-Petrovicova, J. (2015). Literature review on factors influencing milk purchase behavior. *International Review of Management and Marketing, 5* (1), 9-25.

<https://dergipark.org.tr/en/download/article-file/366697>

Laassal, M., & Kallas, Z. (2019). Consumers preferences for dairy-alternative beverage using home-scan data in Catalonia. *Beverages, 5.* https://doi.org/10.3390/beverages5030055

Lautenschlager, L., & Smith, C. (2007). Beliefs, knowledge, and values held by inner-city youth about gardening, nutrition, and cooking. Agriculture and Human Values, 24, 245-258.

Leitzmann, C., & Keller, M., (2013). Vegetarian nutrition. Eugen Ulmer.

Lemke, H. (2011). Climate justice and food culture - or "Learn to love tofu sausages!". In A. Ploeger, G. Hirschfelder & G. Schönberger (Eds.). *The future on the table. Analyses, trends and perspectives of tomorrow's nutrition* (1st ed., pp. 167-186). VS-Verlag & Springer

Lonkila, A., & Kaljonen, M. (2021). Promises of meat and milk alternatives: an integrative literature review on emergent research themes. *Agriculture and Human Values, 38*, 625-639. https://doi.org/10.1007/s10460-020-10184-9

Mäkinen, O. E., Wanhalinna, V., Zannini, E., & Arendt, E. (2016). Foods for special dietary needs: Non-dairy plant based milk substitutes and fermented dairy type products. *Crit. Rev. Food. Scie. Nutr*.

http//dx.doi.org/10.1080/10408398.2012.761950

Markovina, J., Stewart-Knox, B. J., Rankin, A., Gibney, M., de Almeida, M. D. V., Fischer, A., Kuznesof, S. A., Poínhos, R., Panzone, L., & Frewer, L. J. (2015). Food4Me study: validity and reliability of Food Choice Questionnaire in 9 European countries. *Food Quality and Preferences*, *45*, 26-32.

<http://dx.doi.org/10.1016/j.foodqual.2015.05.002>

McCarthy, K. S., Parker, M-, Ameerally, A., Drake, S. L., & Drake, M., A. (2017). Drivers of choice for fluid milk versus plant-based alternatives: what are consumer perceptions of fluid milk? *Journal of Dairy Science, 100,* 6125-6138.

Mendly-Zambo, Z., Powell, L. J., & Newmann, L. L. (2021). Dairy 3.0: cellular agriculture and the future of milk. *Food, Culture & Society. https://doi.org/10.1080/15528014.2021.1888411*

Menzel, W. (1995). Word types. In. Ders (ed.). *Grammar. Practice and background* (14-20). Seelze

Mohler, P., & Wohn, K. (2005). Personal value orientations in the European Social Survey. (ZUMA working report, 2005/01). ZUMA Center for Surveys, Methods and Analysis.

<https://nbn-resolving.org/urn:nbn:de:0168-ssoar-200597>

Palacios, O.M., Badran, J., Spence, L., Drake, M.A., Reisner, M., and Moskowitz, H.R. (2010). Measuring Acceptance of Milk and Milk Substitutes among Younger and Older Children. *Journal of Food Science, 75* (9), 522-526.

Pichler, A. (2009). The health value of milk in the field of tension between scientific discourse and public opinion - a qualitative and quantitative image analysis. Diploma thesis at the University of Natural Resources and Applied Life Sciences in Vienna.

Pieniak, Z., Perez-Cueto, F., & Verbeke, W. (2013). Nutritional status, self-identification as a traditional food consumer and motives for food choice in six European countries. *British Food Journal, 115* (9), 1297-1312. https://doi.org/10.1108/BFJ-08-2011-0198.

Pritulska, N., Motuzka, I., Koshelnyk, A., Motuzka, O., Yashchenko. L., Jarossová, M., Krnáčová, P., Wyka, J., Malczyk, E., & Habánová, M. (2021). Consumer preferences on the market of plant-based milk analogues. Potravinarstvo Slovak Journal of Food Sciences, 15, 131-142. https://doi.org/10.5219/1485

ProVeg (2019). Plant milk report.

https://proveg.com/de/wp-content/uploads/sites/5/2019/10/PV_Pflanzenmilch-Report_281019-final.pdf

Racey, M., Bransfield, J., Capello, K., Field, D., Kulak, V., Machmueller, D., Preyde, M., & Newton, G. (2016). Barriers and Facilitators to Intake of Dairy Products in Adolescent Males and Females With Different Levels of Habitual Intake. *Childhood Obesity and Nutrition, 4.* https://doi.org./10.1177/2333794X17694227

Reyes-Jurado, F., Soto-Reyes, N., Dávila-Rodríguez, M., Lorenzo-Leal, A. C., Jiménez-Munguía, M. T., Mani-López, E., & López-Malo, A. (2021). Plant-Based Milk Alternatives: Types-Processes, Benefits, and Characteristics. *Food Reviews International. https://doi.*org/10.1080/87559129.2021.1952421

Schiano, A. N., Nishku, S., Racette, C. M., & Drake, M. A. (2022). Parent's implicit perceptions of dairy milk and plant-based milk alternatives. Journal of Dairy Science, 105. https//doi.org/10.3168/jds.2021-21626.

Schmidt, P., Bamberg, S., Davidov, E., Hermann, J. & Schwartz, S. H. (2007). Measuring values with the "Portraits Value Questionaire." Journal of Social Psychology, 38 (4), 261-275. <https://doi.org/10.1024/0044-3514.38.4.261>

Schwartz, S. H. (1992). Universals in the content and structure of values: Theoretical advances and empirical tests in 20 countries. *Advances in Experimental Social Psychology, 25.*

Schwartz, S. H. (1999). A Theory of Cultural Values and Some Implications for Work. *Applied Psychology, An International Review, 48* (1), 23-47. <https://doi.org/10.1111/j.1464-0597.1999.tb00047.x>

Schwartz,.S. H. (2021). A Repositiory of Schwartz Value Scales with Instructions and an Introduction. Online Readings in Psychology and Culture, (2). https://doi.[org/10.9707/2307-0919.1173](https://doi.org/10.9707/2307-0919.1173)

Schwartz, S. H., Cieciuch, J., Vecchione, M., Davidoy, E., Fischer, R., Beierlein, C., Ramos, A., Verkasalo, M., Lönnqvist, J.-E., Demirutku, K., Dirilen-Gumus, O., Konty, M. (2012). Refining the Theory of Basic Individual Values. *Journal of Personality and Social Psychology, 103 (4),* 663-688. <https://doi.org/10.1037/a0029393>

Shaw, D., Grehan, E., Shiu, E., Hassan, L., Thomson, J. (2005). An exploration of values in ethical consumer decision making. *Journal of Consumer Behavior, 4 (3)*, 185-200. <https://doi.org/10.1002/cb.3>

Siró, I., Kápolna, E., Kápolna, B., & Lugasi, A. (2008). Functional food. Product development, marketing and consumer acceptance - A review. *Appetite*, 51, 456-467. <https://doi.org/10.1016/j.appet.2008.05.060>

Steptoe, A., Pollard, T. M., Wardle, J. (1995). Development of a measure of the motives underlying the selection of food: the Food Choice Questionnaire. *Appetite, 25* (3), 267-284. <https://doi.org/10.1006/appe.1995.0061>

Szakály, Z., Szente, V., Kövér, G., Polereczki, Z., & Szigeti, O. (2012). The influence of lifestyle on health behavior and preferences for functional foods. *Appetite, 58*, 406-413. https://doi.org/10.1016/j.appet.2011.11.003

Topić, M., & Mitchell, B. (2019). Generation Z and consumer trends in environmental packaging. Project Report. The Retail Institute, Leeds. https://eprints.[leedsbeckett.ac.uk/id/eprint/6066/](https://eprints.leedsbeckett.ac.uk/id/eprint/6066/)

Van den Bergh, J., & Pallini, K. (2018). Marketing to generation Z. *Research World, 70,* 18-23.

<https://doi.org/10.1002/rwm3.20660>

Wikipedia (2022-06-07). Milk substitute. https://de.wikipedia.org/wiki/Milchersatz

Zibenberg, A., Greenspan, I., Katz-Gerro, T., & Handy, F. (2018). Environmental Behavior Among Russian Youth: The Role of Self-direction and Environmental Concern. *Environmental Management, 62*, 295-304. <https://doi.org/10.1007/s00267-018-1032-7>

Zollman Thomas, O., & Dillard, C. (2022). A New Way of Making Dairy: Perceptions, Naming and Implications. https://prismic-io.s3.amazonaws.com/formo/79909028-2cd3-4ab2-8096-1b95f39caf38_Formo_ANewWay_ConsumerReport.pdf

**Materials for the interventions**

**M1: List of motives for/against the consumption of plant-based milk alternatives**

**Consumption of plant-based milk alternatives**

| Consumption |  |
| --- | --- |

| Motives for/against the consumption of plant drinks |
| --- |
|  |
|  |
|  |

| Motives for/against the consumption of plant drinks |
| --- |
|  |
|  |
|  |

### **M2: Information text "animal-free milk”**

**Information about "animal-free milk”**

In Germany, more and more people are reducing their milk consumption and switching to milk alternatives. In addition to plant-based milk alternatives such as oat, soy, or almond milk, "animal-free milk" is another product that could serve as a milk alternative in the future. "Animal-free milk" mainly consists of water and cow's milk proteins. The milk proteins are responsible for the taste of cow's milk. To produce "animal-free milk", the cow's milk proteins are not produced by cows, but by microorganisms such as bacteria or yeasts. For this purpose, the cow's DNA sequences, which code for the milk proteins, are integrated into the microorganisms' DNA. The microorganisms are multiplied in a suitable culture medium and produce the cow's milk proteins. These can be further processed into "animal-free milk" or "animal-free milk"-products such as yogurt, cream cheese, or ice cream. The first animal-free dairy products have been available on the US market since 2021.

### **Material M3: List of motives for/against the consumption of "animal-free milk**

**Willingness to consume "animal-free milk”**

| Willingness to consume |  |
| --- | --- |

| Motives for/against the willingness to consume "animal-free milk |
| --- |
|  |
|  |
|  |

| Motives for/against the willingness to consume "animal-free milk |
| --- |
|  |
|  |
|  |

**M4: Interview questionnaire**

**Part 1** (to be filled in by the interviewee)

| **Interview No. (Anonymized Name)** |  |
| --- | --- |

| **Attitudes towards plant-based milk alternatives** |
| --- |

**Plant-based milk alternatives are...**

|  | -3 | -2 | -1 | 0 | +1 | +2 | +3 |  |
| --- | --- | --- | --- | --- | --- | --- | --- | --- |
| disgusting |  |  |  |  |  |  |  | tasty |
| unhealthy |  |  |  |  |  |  |  | healthy |
| dirty |  |  |  |  |  |  |  | clean |
| primitive |  |  |  |  |  |  |  | civilized |
| nutrient-poor |  |  |  |  |  |  |  | nutrient-rich |
| unsustainable |  |  |  |  |  |  |  | sustainable |
| uninteresting |  |  |  |  |  |  |  | interesting |
| not promising |  |  |  |  |  |  |  | promsing |
| artificial |  |  |  |  |  |  |  | natural |
| old-fashioned |  |  |  |  |  |  |  | modern |
| ethically questionable |  |  |  |  |  |  |  | ethically not questionable |

**In general, I evaluate plant-based milk alternatives as...**

|  | -3 | -2 | -1 | 0 | +1 | +2 | +3 |  |
| --- | --- | --- | --- | --- | --- | --- | --- | --- |
| negative |  |  |  |  |  |  |  | positive |

| **Willingness to consume plant-based milk alternatives** |
| --- |

| **How likely are you to try plant-based milk alternatives?** | | | | |
| --- | --- | --- | --- | --- |
| very  unlikely |  |  |  | very likely |
| -2 | -1 | 0 | +1 | +2 |
|  |  |  |  |  |

| **How likely are you to buy plant-based milk alternatives?** | | | | |
| --- | --- | --- | --- | --- |
| very  unlikely |  |  |  | very likely |
| -2 | -1 | 0 | +1 | +2 |
|  |  |  |  |  |

| **How likely is it that you would use plant-based milk alternatives as a substitute for cow's milk?** | | | | |
| --- | --- | --- | --- | --- |
| very  unlikely |  |  |  | very likely |
| -2 | -1 | 0 | +1 | +2 |
|  |  |  |  |  |

| **Attitudes towards "animal-free milk** |
| --- |

**"animal-free milk is...**

|  | -3 | -2 | -1 | 0 | +1 | +2 | +3 |  |
| --- | --- | --- | --- | --- | --- | --- | --- | --- |
| disgusting |  |  |  |  |  |  |  | tasty |
| unhealthy |  |  |  |  |  |  |  | healthy |
| dirty |  |  |  |  |  |  |  | clean |
| primitive |  |  |  |  |  |  |  | civilized |
| nutrient-poor |  |  |  |  |  |  |  | nutrient-rich |
| unsustainable |  |  |  |  |  |  |  | sustainable |
| uninteresting |  |  |  |  |  |  |  | interesting |
| not promising |  |  |  |  |  |  |  | promsing |
| artificial |  |  |  |  |  |  |  | natural |
| old-fashioned |  |  |  |  |  |  |  | modern |
| ethically questionable |  |  |  |  |  |  |  | ethically not questionable |

**In general, I evaluate "animal-free milk" as...**

|  | -3 | -2 | -1 | 0 | +1 | +2 | +3 |  |
| --- | --- | --- | --- | --- | --- | --- | --- | --- |
| negative |  |  |  |  |  |  |  | positive |

| **Willingness to consume "animal-free milk** |
| --- |

| **How likely is it that you would try "animal-free milk"?** | | | | |
| --- | --- | --- | --- | --- |
| very  unlikely |  |  |  | very likely |
| -2 | -1 | 0 | +1 | +2 |
|  |  |  |  |  |

| **How likely is it that you would buy "animal-free milk"?** | | | | |
| --- | --- | --- | --- | --- |
| very  unlikely |  |  |  | very likely |
| -2 | -1 | 0 | +1 | +2 |
|  |  |  |  |  |

| **How likely is it that you would use "animal-free milk" as a substitute for cow's milk?** | | | | |
| --- | --- | --- | --- | --- |
| very  unlikely |  |  |  | very likely |
| -2 | -1 | 0 | +1 | +2 |
|  |  |  |  |  |

| **Sociodemographic and nutritional data** | | | | |
| --- | --- | --- | --- | --- |
| **Date** |  | | | |
| **School** (room or place of the interview) |  | | | |
| **Time** (duration) |  | | | |
| **Gender** |  | | | |
| **Age** |  | | | |
| **Residence** | rather close to the city | | rather rural | |
| **Diet** | omnivor | vegetarian | vegan | different |
| **Co-determination purchase of food** | rather yes | | rather no | |
| **Consumption plant-based milk alternatives** | daily | weekly | monthly | never |
| **Consumption cow's milk** | daily | weekly | monthly | never |

**Part 2** (to be completed by the interviewer)

| **Interview specific data** | |
| --- | --- |
| **Anomalies**  (external conditions) |  |
| **Impression of the subject**  (personality, mood, appearance) |  |
| **Atmosphere during the interview** |  |
| **Interruptions/**  **Interference** |  |
| **Spontaneity of associations** (comprehensive, etc.) |  |
| **Other** |  |
